# Dynamic m^6^A mRNA methylation reveals the role of METTL3-m^6^A-CDCP1 signaling axis in chemical carcinogenesis

**DOI:** 10.1038/s41388-019-0755-0

**Published:** 2019-02-22

**Authors:** Fan Yang, Huan Jin, Biao Que, Yinghui Chao, Haiqing Zhang, Xiaoling Ying, Zhongyang Zhou, Zusen Yuan, Jialin Su, Bin Wu, Wenjuan Zhang, Defeng Qi, Demeng Chen, Wang Min, Shuibin Lin, Weidong Ji

**Affiliations:** 1 Center for Translational Medicine, The First Affiliated Hospital, Sun Yat-sen University, Guangzhou, 510080 China; 2 Department of Physiology, Zunyi Medical College, Guizhou, 563000 China; 3 Guangdong Key Laboratory of Urology, Department of Urology, Minimally Invasive Surgery Center, The First Affiliated Hospital of Guangzhou Medical University, Guangzhou, Guangdong 510230 China; 4 The Second Affiliated Hospital of Guangzhou University of Chinese Medicine, Guangzhou, 510080 China; 5 Department of Preventive Medicine, The School of Medicine, Jinan University, Guangzhou, 510632 China; 6 Department of Pathology and the Vascular Biology and Therapeutics Program, Yale University School of Medicine, New Haven, CT 06519 USA

**Keywords:** Oncogenes, Bladder cancer, Gene silencing

## Abstract

N6-methyladenosine (m^6^A) is the most abundant internal modification in mammalian mRNAs. Despite its functional importance in various physiological events, the role of m^6^A in chemical carcinogenesis remains largely unknown. Here we profiled the dynamic m^6^A mRNA modification during cellular transformation induced by chemical carcinogens and identified a subset of cell transformation-related, concordantly modulated m^6^A sites. Notably, the increased m^6^A in 3′-UTR mRNA of oncogene CDCP1 was found in malignant transformed cells. Mechanistically, the m^6^A methyltransferase METTL3 and demethylases ALKBH5 mediate the m^6^A modification in 3′-UTR of CDCP1 mRNA. METTL3 and m^6^A reader YTHDF1 preferentially recognize m^6^A residues on CPCP1 3′-UTR and promote CDCP1 translation. We further showed that METTL3 and CDCP1 are upregulated in the bladder cancer patient samples and the expression of METTL3 and CDCP1 is correlated with the progression status of the bladder cancers. Inhibition of the METTL3-m^6^A-CDCP1 axis resulted in decreased growth and progression of chemical-transformed cells and bladder cancer cells. Most importantly, METTL3-m^6^A-CDCP1 axis has synergistic effect with chemical carcinogens in promoting malignant transformation of uroepithelial cells and bladder cancer tumorigenesis in vitro and in vivo. Taken together, our results identify dynamic m^6^A modification in chemical-induced malignant transformation and provide insight into critical roles of the METTL3-m^6^A-CDCP1 axis in chemical carcinogenesis.

## Introduction

The dynamic modifications on mRNAs are essential for posttranscriptional regulation of gene expression [1, 2]. N6-methyladenosine (m^6^A), the most abundant internal mRNA modification, has important roles in regulating mRNA splicing, export, stability, and translation [1, 2]. m^6^A is catalyzed by an RNA methyltransferase complex consisting of the catalytic subunit METTL3 and its assisting partners METTL14 and WTAP [3, 4]. On the other hand, two demethylases, ALKBH5 and FTO, are responsible for the removal of m^6^A from mRNAs [5, 6]. Several different m^6^A reader proteins, including the YTHDF and YTHDC family members, can specifically recognize m^6^A modification and regulate the processing, degradation, and translation of m^6^A-modified mRNAs [7–11]. The dynamic interplay between m^6^A pathway components cooperatively regulates fine-turning mRNA metabolism and translation.

Emerging in vitro and in vivo studies uncovered the critical roles of m^6^A modification in regulation of diverse biological processes including spermatogenesis [6], neurogenesis [12], sex determination [13, 14], stem cell self-renewal, and fate determination [15–17]. Moreover, mis-regulations of m^6^A pathway are frequently found in cancers such as lung cancer [18], breast cancer [19], glioblastomas [20–22], acute myeloid leukemia [23, 24], and hepatocellular carcinoma [25]. Interestingly, depending on the molecular targets, the m^6^A modification pathway could have oncogenic or tumor suppressive functions in different cellular contexts [18–25]. Gain-of-function and loss-of-function studies revealed that aberrant m^6^A modification is essential for cancer growth and progression [18–25], suggesting that the m^6^A modification pathway could be a promising therapeutic target for cancer therapy.

Chemical carcinogenesis is a multistage process that leads to the malignant tumor formation in animals and humans [26]. Chemical carcinogens can bind to DNA and induce genetic mutations in cancer-susceptibility genes, resulting in the development of carcinogenesis [27]. Importantly, exposure to chemical carcinogens also leads to epigenetic changes such as aberrant DNA methylation or histone modifications, which coordinate with the genetic mutations to initiate oncogenesis [27, 28]. The m^6^A modification pathway is frequently mis-regulated in cancers; however, the role of m^6^A modification in chemical-induced cellular transformation remains unknown.

Here we studied the functions and the underlying molecular mechanisms of m^6^A mRNA modification in chemical carcinogenesis. We found that chemical carcinogen treatment induces dynamic changes of m^6^A modification and identified oncogene CDCP1 as a critical m^6^A target in chemical-induced transformation. We further showed that the METTL3-m^6^A-CDCP1 axis interplays with chemical carcinogens to promote the malignant transformation. Our data uncovered novel epitranscriptomic mechanisms in chemical carcinogenesis.

## Results

### Topology of m^6^A mRNA methylome during cell malignant transformation

**Fig. 1:**
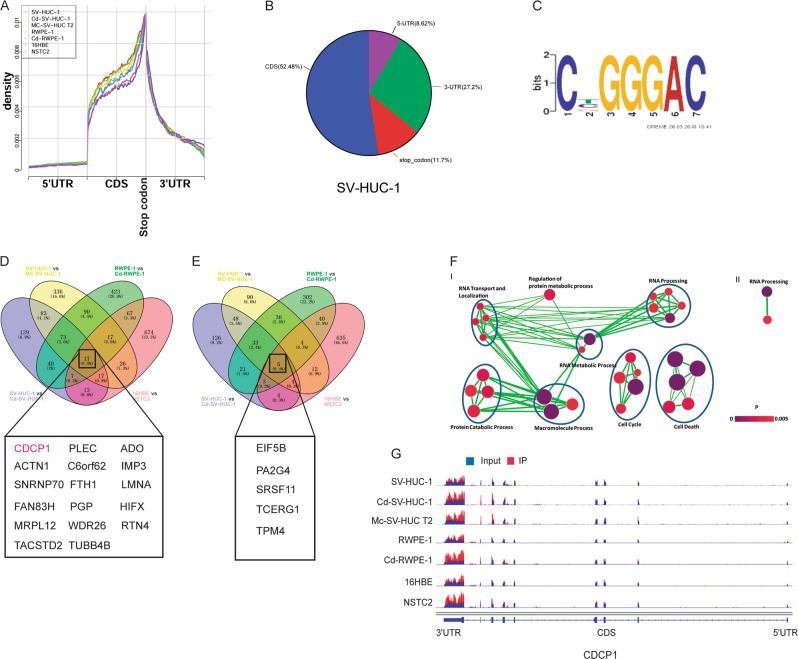
Dynamic m^6^A modification patterns during chemical-induced cells transformation. **a** Metagene profiles of m^6^A distribution across the transcriptome in control and carcinogen induced cells. **b** Distribution of m6A peaks in the 5′-UTR (pink), coding sequence (CDS, blue), stop code(green), and 3′-UTR (red) of RNA transcripts. Representative charts show the proportion of m^6^A peaks in the indicated regions in SV-HUC-1 cells. **c** Consensus sequence motif for m^6^A methylation identified in SV-HUC-1 cells. **d** Overlap of genes with upregulated m^6^A modifications in the chemical-transformed cells. **e** Overlap of genes with downregulated m^6^A modifications in the transformed cells. **f** Gene Ontology Biological Process (GOBP) and Enrichment Analysis of genes with differential m^6^A modifications in control SV-HUC-1 vs. Cd, MCA-induced SV-HUC-1 transformed cells. (I) Analysis using 190 genes with upregulated m^6^A peaks in both Cd and MCA-induced transformed cells; (II) analysis using 90 genes with downregulated m^6^A peaks in both Cd and MCA-induced transformed cells. Each corresponding set of targets was subjected to DAVID GOBP analysis and an enrichment map was built by Cytoscape with Enrichment Map Apps. Each node denotes one enriched GOBP pathway (*p* < 0.005, FDR *q* < 0.1, overlap cutoff > 0.5), with its color reflecting the *p*-value. Node size is proportional to the total number of genes in each pathway. GOBP pathways with similar function were sorted into one cluster, marked with circles and labels. G representative differentially regulated m^6^A of CDCP1 mRNA in chemical transformation; IP immunoprecipitation

To study the role of m^6^A pathway during cell malignant transformation, we first performed the transcriptome-wide profiling of m^6^A modification using the chemical carcinogen-induced transformation model. The human uroepithelial SV-HUC-1 cells, human prostate epithelial RWPE-1 cells, and human bronchial epithelial 16HBE cells were treated with chemical carcinogens (Cadmium (Cd), 3-methylcholanthrene, and Nickel) to induce malignant transformation [29, 30] (Figure S1). Then total RNA samples from the normal and transformed cells were isolated for m^6^A profiling using m^6^A methylated RNA immunoprecipitation sequencing (MeRIP-Seq). Metagene and distribution analysis of the m^6^A modification revealed that m^6^A peaks are enriched mostly in CDS and 3′-intranslated region (UTR) region (Fig. 1b). The density of m^6^A peaks increases steadily along transcript in CDS. Conversely, in the 3′-UTR, the peaks decrease in abundance along the length of the 3′-UTR (Fig. 1a). Comparison of m^6^A distribution between normal and malignant cells revealed that chemical treatment has little effect on the m^6^A distribution in mRNAs (Fig. 1a). Motif searching identified the consensus “GGAC” motif within the m^6^A sites (Fig. 1c). Overall, our m^6^A profiling results are consistent with the published m^6^A features, suggesting that we have successfully identified the specific m^6^A sites in the control and malignant cells.

### Identification of chemical transformation-induced m^6^A targets

We next investigated the differentially modified targets in the normal and transformed cells with m^6^A MeRIP-Seq data from four different sets of comparable cells: SV-HUC-1 control and Cd-transformed cells (SV-HUC-1 vs. Cd-SV-HUC-1), SV-HUC-1 control and 3-methylcholanthrene-transformed cells (SV-HUC-1 vs. MC-SV-HUC-1), RWPE-1 control and Cd-transformed cells (RWPE-1 vs. Cd- RWPE-1), and 16HBE control and Nickel-transformed cells (16HBE vs. NSTC2). As shown in Fig. 1d, e, chemical transformation induced dynamic changes of m^6^A modifications in hundreds of mRNAs in each set of control and transformed cells. Overlapping of the upregulated (Fig. 1d) and downregulated (Fig. 1e) sites revealed the dynamic and diverse m^6^A changes in different chemical-mediated transformation models. Gene ontology analysis revealed that the common chemical transformation induced m^6^A gene function in regulation of RNA processing and metabolic process, protein process, cell cycle, and cell death. On the other hand, the common genes with decreased m^6^A modifications in the transformed cells function in RNA processing regulation (Fig. 1f). Comparison of m^6^A profiles in control and transformed cells identified many cancer-promoting genes as m^6^A targets during chemical carcinogenesis. For example, the m^6^A modification on the mRNA of oncogene CDCP1 was upregulated in all four transformation models (Fig. 1g), suggesting the potential role of chemical-induced m^6^A modification of CDCP1 in regulation of oncogenic transformation.

### CDCP1 mRNA translation, but not degradation, was mediated by the m^6^A modification

**Fig. 2:**
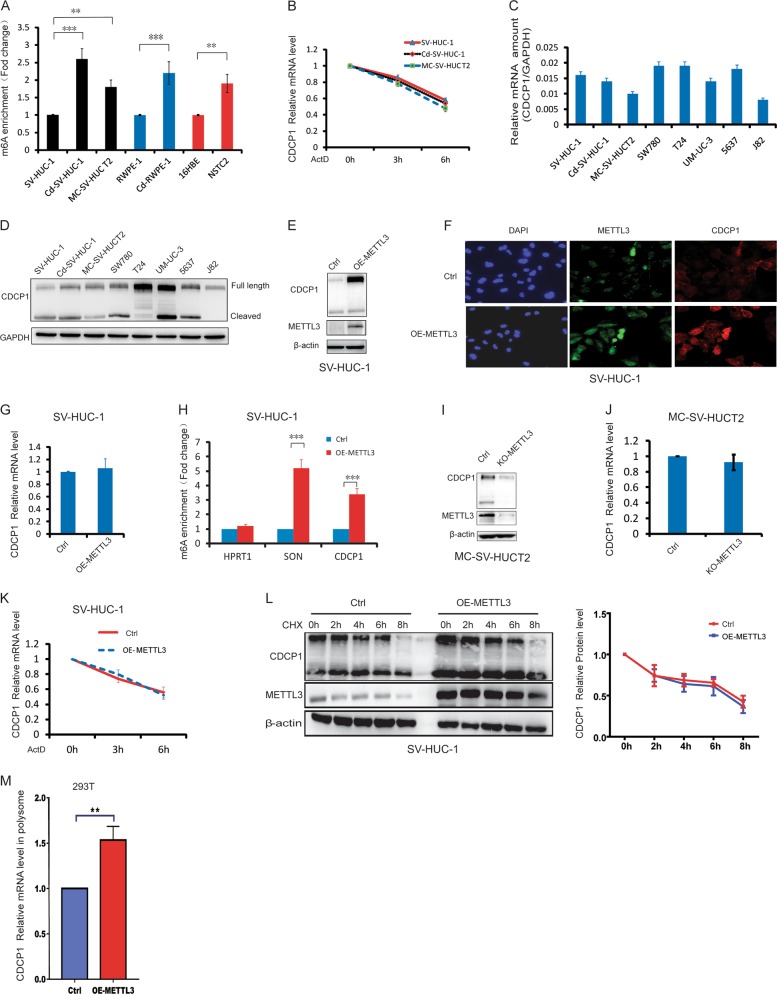
METTL3 promotes CDCP1 mRNA m^6^A modification and translation. **a** m^6^A enrichment of CDCP1 mRNA 3′-UTR in chemical-transformed cells vs. control cells was validated by MeRIP-qRT-PCR. **b** CDCP1 mRNA stability was determined by qRT-PCR in control and transformed SV-HUC-1 cells using samples treated with ActD at the indicated times. Error bars, mean ± SEM; *n* = 3 biological replicates. **c** qRT-PCR analysis of CDCP1 mRNA expression in SV-HUC-1 cells, transformed cells (Cd-SV-HUC-1, MC-SV-HUC T2), and human bladder cancer cells (SW780, T24, UM-UC-3, 5637, J82). GAPDH was used as an internal control. **d** Western blotting of CDCP1 in SV-HUC-1 cells, transformed cells (Cd-SV-HUC-1, MC-SV-HUCT2), and human bladder cancer cells (SW780, T24, UM-UC-3, 5637, J82). **e** Western blotting of CDCP1 protein expression in control and stable METTL3-overexpressing SV-HUC-1 cells (OE-METTL3 SV-HUC-1). **f** Immunofluorescence analysis of CDCP1 expression in control and OE-METTL3 SV-HUC-1 cells. **g** qRT-PCR analysis of CDCP1 mRNA expression in control and OE-METTL3 SV-HUC-1 cells. **h** m^6^A enrichment of CDCP1 mRNA 3′-UTR in control and OE-METTL3 SV-HUC-1 cells. SON, positive control; HPRT1, negative control. **i** Western blotting of CDCP1 in control and METTL3-depleted MC-SV-HUCT2 cells (KO-METTL3 MC-SV-HUCT2). **j** qRT-PCR analysis of CDCP1 mRNA expression in control and KO-METTL3 MC-SV-HUCT2 cells. **k** CDCP1 mRNA stability in control and OE-METTL3 SV-HUC-1 cells. **l** CDCP1 protein stability in control and OE-METTL3 SV-HUC-1 cells. Left panel, representative images of METTL3 and CDCP1 western blotting. Right panel, the relative expression of CDCP1 protein in a time course manner after CHX treatment. **m** CDCP1 mRNA expression of polysomal fractionated RNA in control and METTL3-overexpressing 293T cells. All bar plot data are means ± SEM of three independent experiments except **h**, where error bars denote SD of technical triplicates. **p < 0.01, ***p < 0.001

The dysregulated expression of oncogene CDCP1 promotes cancer transformation and progression in vitro and in vivo [31]. Therefore, we further studied the molecular mechanisms and functions of CDCP1 m^6^A modification in chemical transformation. m^6^A MeRIP-qRT-PCR (quantitative reverse-transcriptase PCR) confirmed that the CDCP1 m^6^A modification is significantly increased upon chemical transformation (Fig. 2a), whereas the CDCP1 mRNA stability remained unchanged (Fig. 2b). We further compared the CDCP1 mRNA and protein levels in the chemical-transformed cells and other bladder cancer cells. Interestingly, CDCP1 protein expression, but not its mRNA, is upregulated in transformed cells and bladder cancer cells (Fig. 2c, d). Overall, these data suggested the potential posttranscriptional regulation of CDCP1 expression through m^6^A modifications during chemical carcinogenesis.

### The m^6^A methyltransferase METTL3 promotes CDCP1 mRNA modification and translation

The m^6^A methyltransferase METTL3 catalyzes m^6^A modifications to facilitate target mRNA degradation or translation. Given that CDCP1 mRNA stability and level remained unchanged but CDCP1 protein increased in the chemical-transformed cells, we tested the possibility of METTL3-mediated m^6^A modification in regulation of CDCP1 mRNA translation. Overexpression of METTL3 in SV-HUC-1 increased the CDCP1 protein level (Fig. 2e, f) but had little effect on CDCP1 mRNA (Fig. 2g). METTL3 overexpression significantly promoted the m^6^A modification in CDCP1 mRNA and the mRNA of known m^6^A target SON, but not the negative control HPRT1 mRNA (Fig. 2h), suggesting that METTL3-mediated m^6^A modification promotes the CDCP1 protein expression. On the other hand, depletion of METTL3 in the transformed MC-SV-HUC-1 cells and bladder cancer T24 cells resulted in decreased level of CDCP1 protein but not the mRNA (Fig. 2i, j, S2A, S2B). Moreover, similar to chemical transformation, METTL3 overexpression has little effect in the stability of CDCP1 mRNA (Fig. 2k). As controls, the stability of m^6^A containing SON mRNA is significantly downregulated in the METTL3-overexpressing cells (Figure S3A), whereas the stability of the m^6^A-negative mRNA HPRT1 remain unchanged (Figure S3B). To rule out potential effects of METTL3 on CDCP1 protein stability, we treated cells with cycloheximide (CHX) to block translation and measured CDCP1 degradation. Degradation rates of CDCP1 protein were significantly unchanged between control and METTL3-overexpressing cells (Fig. 2l). Furthermore, polysome-bound (translationally active) CDCP1 mRNA levels significantly increased in METTL3-overexpressing cells compared with control cells (Fig. 2m). In contrast, depletion of METTL3 in T24 cells significantly reduced the proportion of CDCP1 transcripts in polysomal fractions (Figure S2C), suggesting that METTL3 enhances CDCP1 mRNA translation. Overall, our data indicated that METTL3 catalyzes the m^6^A modification on CDCP1 mRNA and promotes its translation upon chemical transformation.

### m^6^A demethylase ALKBH5 regulates CDCP1 mRNA modification and translation

**Fig. 3:**
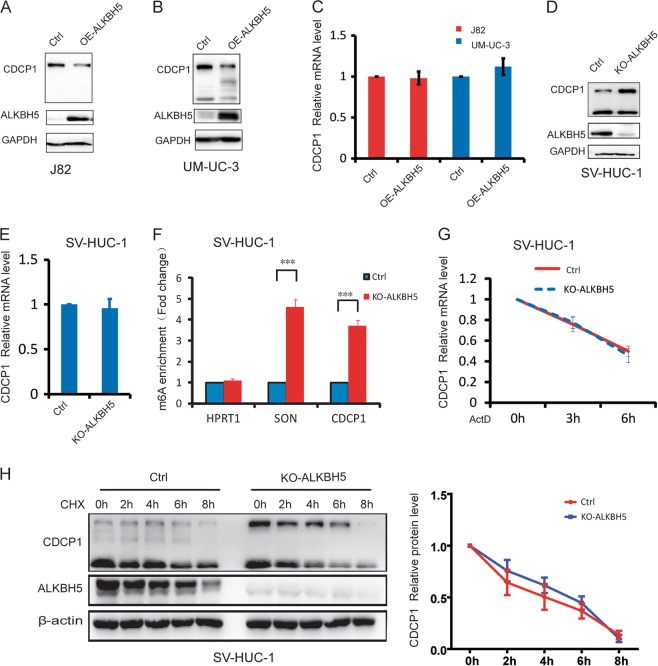
m^6^A demethylase ALKBH5 regulates CDCP1 mRNA modification and translation. **a** Western blotting of CDCP1 in control and ALKBH5-overexpressing J82 cells (OE-ALKBH5 J82). **b** Western blotting of CDCP1 in control and ALKBH5-overexpressing UM-UC-3 cells (OE-ALKBH5 UM-UC-3). **c** qRT-PCR analysis of CDCP1 mRNA expression in control, OE-ALKBH5 J82, and OE-ALKBH5 UM-UC-3 cells. **d** Western blotting of CDCP1 in control and ALKBH5-depletion cells (KO-ALKBH5 SV-HUC-1). **e** qRT-PCR analysis of CDCP1 mRNA expression in control and KO-ALKBH5 SV-HUC-1 cells. **f** m^6^A enrichment of CDCP1 mRNA 3′-UTR in control and KO-ALKBH5 SV-HUC-1 cells. SON, positive control; HPRT1, negative control. **g** CDCP1 mRNA stability in control and KO-ALKBH5 SV-HUC-1 cells. **h** CDCP1 protein stability in control and KO-ALKBH5 SV-HUC-1 cells. Left panel, representative images of METTL3 and CDCP1 western blotting. Right panel, the relative expression of CDCP1 protein in a time course manner after CHX treatment. All bar plot data are means ± SEM of three independent experiments except **f**, where error bars denote SD of technical triplicates. *p < 0.05, **p < 0.01, ***p < 0.001

To study the functions and mechanisms of m^6^A modification in regulation of CDCP1 expression during chemical carcinogenesis, we determined the roles of m^6^A demethylases, ALKBH5 and FTO, in regulation of CDCP1 expression. Overexpression of ALKBH5 in bladder cancer J82 cells and UM-UC-3 cells resulted in decreased CDCP1 protein level without changing its mRNA (Fig. 3a–c). On the other hand, depletion of ALKBH5 increased level of CDCP1 proteins but not the mRNA (Fig. 3d, e). Interestingly, overexpression or depletion of FTO did not change CDCP1 expression (Figure S4A-S4D), suggesting that ALKBH5 and FTO might have different molecular targets. Consistent with ALKBH5’s function as an m^6^A demethylase, ALKBH5 depletion resulted in increased m^6^A modification on CDCP1 mRNA (Fig. 3f). Moreover, manipulation of ALKBH5 expression did not significantly change the stability of CDCP1 mRNA and protein (Fig. 3g, h). Overall, ALKBH5 demethylates CDCP1 mRNA and negatively regulates CDCP1 protein expression, further supporting the role of m^6^A pathway in regulation of CDCP1 mRNA modification and translation.

### m^6^A motifs within the 3′-UTR of CDCP1 facilitate the translation of CDCP1 mRNA

**Fig. 4:**
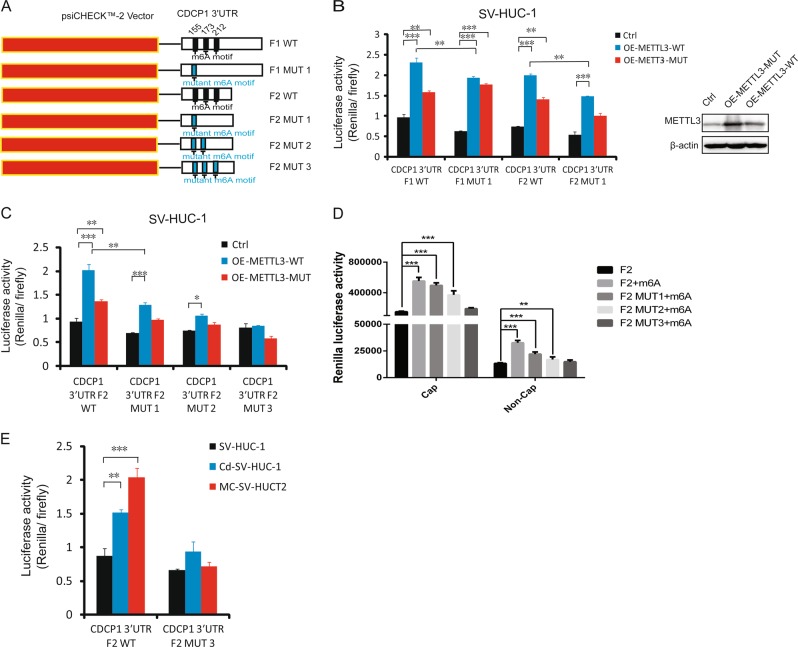
m^6^A motifs within the 3′-UTR of CDCP1 facilitate the translation of CDCP1 mRNA. **a** psiCHECK™-2 luciferase reporter constructs containing two fragments of human CDCP1 3′-UTR that have three putative m6A motifs (F1 WT, F2 WT) or mutant (A-to-T mutation) m^6^A sites (F1 MUT1, F2 MUT1, F2 MUT2, F2 MUT3) are shown. The position of m^6^A sites (155, 173, 212) was numbered relative to the first nucleotide of the 3′-UTR. **b** Relative luciferase activities of psiCHECK™-2- CDCP1 3′-UTR with either wild-type (F1 WT, F2 WT) or one mutant m^6^A sites (F1 MUT1, F2 MUT1) in stable METTL3-overexpressing (OE-METTL3-WT), METTL3 mutant-overexpressing (OE-METTL3-MUT), and control SV-HUC-1 cells. *Renilla* luciferase activities were measured and normalized to Firefly luciferase activity. **c** Relative luciferase activity of psiCHECK™-2- CDCP1 3′-UTR with either F2 wild-type (F2 WT) or 1,2,3 mutant m^6^A sites (F2 MUT1, F2 MUT2, F2 MUT3) in control and OE-METTL3-WT, OE-METTL3-MUT SV-HUC-1 cells. **d** *Renilla* luciferase activity was translated in vitro using Flexi Rabbit Reticulocyte Lysate System. *Renilla* luciferase reporter mRNAs with CDCP1 3′-UTR (F2 WT, F2 MUT1, F2 MUT2, F2 MUT3) was transcribed in vitro in the absence or presence of m^6^A, followed by addition of a function cap m7GpppG or a non-functional cap analog ApppG. **e** Relative luciferase activity of psiCHECK™-2- CDCP1 3′-UTR with either F2 wild-type (F2 WT) or three mutant m6A sites (F2 MUT3) in SV-HUC-1 cells, transformed cells (Cd-SV-HUC-1, MC-SV-HUC T2). All bar plot data are means ± SEM of three independent experiments. *p < 0.05, **p < 0.01,***p < 0.001

To explore the molecular mechanisms underlying the m^6^A-mediated posttranscriptional regulation of CDCP1 in chemical transformation, we first analyzed the CDCP1 mRNA sequence and found three m^6^A motifs within the m^6^A peak region located in the 3′-UTR of CDCP1. We then cloned the CDCP1 3′-UTR region into a luciferase reporter to determine the function of these m^6^A motifs in regulation of gene expression. As shown in Fig. 4a, b, overexpression of METTL3 promoted the translation of luciferase reporters with longer region (F1 WT) or shorter region (F2 WT) of CDCP1 3′-UTR. Mutation of the first m^6^A motif (F1 MUT1, F2 MUT1) slightly decreased METTL3’s function in promoting translation (Fig. 4b), whereas mutation of the three motifs (F2 MUT3) completely abolished the translation enhancement driven by METTL3 (Figure. 4c, S5), suggesting that the three m^6^A motifs are essential for METTL3 to regulate CDCP1 expression. Interestingly, the catalytic mutant METTL3 (METTL3 MUT) can also promote the translation of CDCP1 reporters, although with weaker activity compared with the METTL3 wild type (WT) (Fig. 4b, c, S5), which is consistent with the previous report that the N-terminal METTL3 is sufficient to promote translation when tethered to target mRNAs [18]. In vitro translation using the control and m^6^A-modified reporter mRNAs further confirmed the role of m^6^A modification in promoting CDCP1 translation (Fig. 4d). Most importantly, transfection of the CDCP1 reporters into the control and chemical-transformed cells revealed that chemical-induced transformation significantly increased the WT CDCP1 reporter expression but not the m^6^A motif-mutated reporter (Fig. 4e). Overall, the above results supported that the chemical transformation induces the m^6^A modification of CDCP1 3′-UTR and promotes the translation of CDCP1 mRNA.

### METTL3/YTHDF1 preferentially recognizes m^6^A residues on CDCP1 3′-UTR

**Fig. 5:**
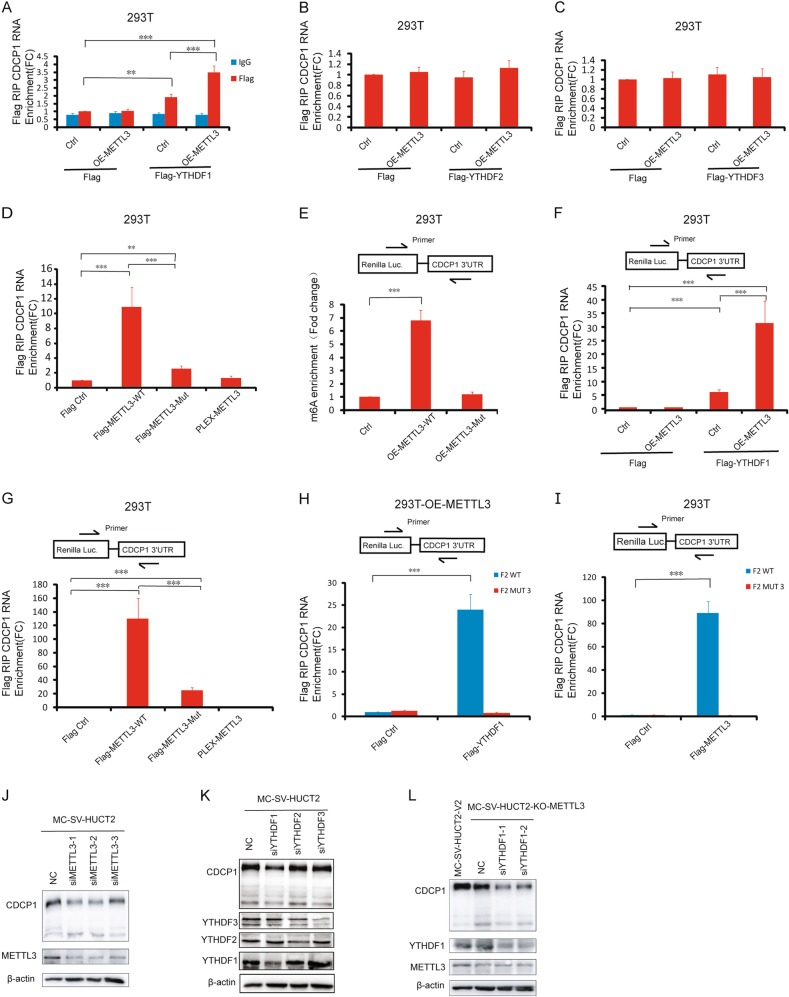
METTL3 and YTHDF1 recognize m^6^A residues on CPCP1 3′-UTR and regulate CDCP1 expression. **a**, **b**, **c** RIP analysis of binding of YTHDF1, YTHDF2, YTHDF3 proteins to CDCP1 mRNA. Both stable METTL3-overexpressing (OE-METTL3) and control 293T cells were transfected with FLAG-tagged YTHDF1, YTHDF2, YTHDF3, or control 2AB vectors. Lysates were immunoprecipitated with an anti-FLAG antibody. CDCP1 mRNA in YTHDF1, YTHDF2, YTHDF3 immunoprecipitates was quantified by qRT-PCR and normalized to the level in 293T control cells expressing 2AB vector. *N* = 3. All data are the mean ± SEM of the indicated number of replicates. **p < 0.01, ***p < 0.001. **d** RIP analysis of binding of METTL3 proteins to CDCP1 mRNA. 293T cells were transfected with FLAG-tagged METTL3 WT, METTL3 MUT, non-FLAG-tagged METTL3 WT, or control 2AB vectors. CDCP1 mRNA in METTL3 immunoprecipitates was quantified by qRT-PCR and normalized to the level in 293T cells expressing 2AB vector. **e** qPCR analysis of m6A levels in exogenous *Renilla* Luc-CDCP1 3′-UTR mRNA in OE-METTL3-WT, OE-METTL3 MUT 293T cells, and 293T control cells. Primer covers the joint of *Renilla* Luc and CDCP1 3′-UTR. **f** RIP analysis of binding of YTHDF1 protein to exogenous CDCP1 mRNA 3′-UTR in OE-METTL3 and control 293T cells. **g** RIP analysis of binding of METTL3 proteins to exogenous CDCP1 mRNA 3′-UTR. **h** RIP analysis of binding of YTHDF1 protein to exogenous CDCP1 mRNA 3′-UTR containing m^6^A sites (F2 WT) and mutant 3 m^6^A sites (F2 MUT3). **i** RIP analysis of binding of METTL3 proteins to exogenous CDCP1 mRNA 3′-UTR containing m^6^A sites (F2 WT) and mutant 3 m^6^A sites (F2 MUT3). **j** Western blotting of CDCP1 expression in MC-SV-HUC T2 cells treated with control or METTL3 siRNAs. **k** Western blotting of CDCP1 expression in MC-SV-HUC T2 cells treated with control or YTHDF1, YTHDF2, YTHDF3 siRNAs. **l** Western blotting of CDCP1 expression in MC-SV-HUC T2-KO-METTL3 cells treated with control or YTHDF1 siRNAs. All bar plot data are means ± SEM of three independent experiments except **e**, where error bars denote SD of technical triplicates. *p < 0.05, **p < 0.01, ***p < 0.001

We next sought to understand how the m^6^A machinery promotes CDCP1 translation upon chemical transformation. METTL3 and other m^6^A readers YTHDF1, YTHDF2, and YTHDF3 selectively recognize m^6^A modification and have important roles in regulation of m^6^A target gene expression. Therefore, we first tested whether YTHDFs bind to CDCP1 mRNA. RNA immunoprecipitation assay revealed that YTHDF1, but not YTHDF2 or YTHDF3, selectively binds to CDCP1 mRNA (Fig. 5a–c, S6A). Moreover, forced expression of METTL3 significantly increased the interaction between YTHDF1 and CDCP1 mRNA, suggesting that METTL3 mediates the m^6^A modification and facilitates YTHDF1 binding to CDCP1 mRNA (Fig. 5a–c, S6B). WT METTL3 protein binds to CDCP1 mRNA and, interestingly, the catalytic mutant METTL3 also interacts with the endogenous CDCP1 mRNA, although with less binding capacity compared with WT (Fig. 5d, S6V, S6D). Using the CDCP1 reporter, we found that only the WT METTL3 can promote the m^6^A modification of CDCP1 reporter mRNA, whereas the METTL3 MUT has no catalytic activity (Fig. 5e). Similar to the findings in the endogenous CDCP1 mRNA, both METTL3 WT and MUT proteins interact with CDCP1 reporter mRNA and overexpression of METTL3 promotes the binding of YTHDF1 to CDCP1 reporter mRNA (Fig. 5f, g). Consistent with the functional assay (Fig. 4c), METTL3 and YTHDF1 only interact with the WT CDCP1 reporter but not the m^6^A motif mutated reporter (Fig. 5h, i, S6E, S6F). In addition, inhibition of METTL3 or YTHDF1, but not YTHDF2/3 expression, decreased CDCP1 expression (Fig. 5j, k) and knocking down both METTL3 and YTHDF1 had synergistic effect in reducing CDCP1 expression. Taken together, our data revealed that METTL3 and YTHDF1 bind to the m^6^A motifs in the CDCP1 3′-UTR and promotes CDCP1 mRNA modification and translation.

### METTL3 and CDCP1 are upregulated in bladder cancers and correlated with bladder cancer progression

**Fig. 6:**
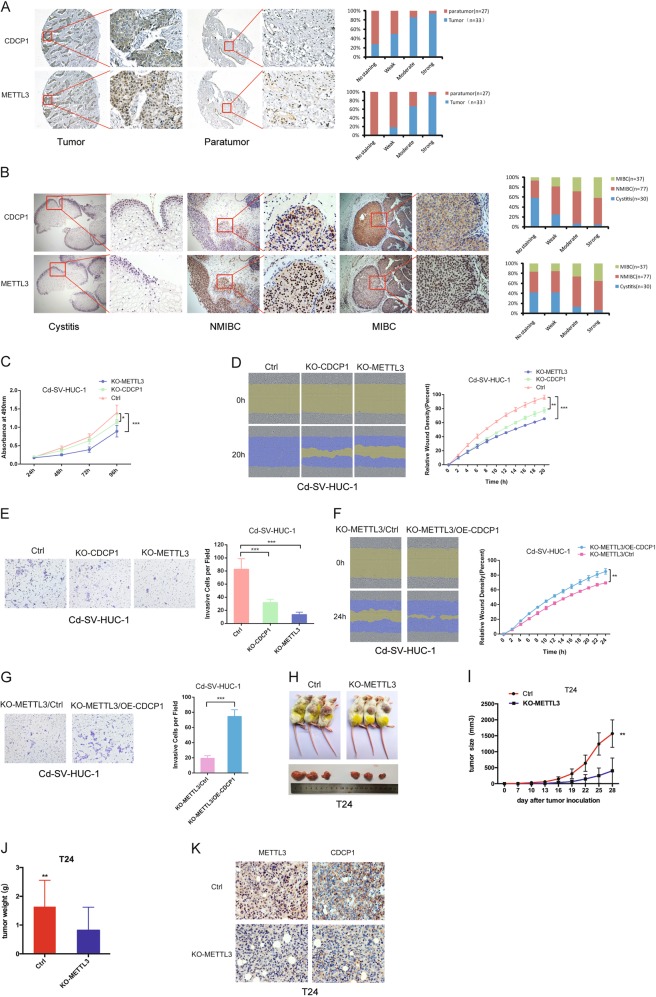
METTL3 and CDCP1 are upregulated in bladder cancers and facilitate bladder cancer progression. **a** METTL3 and CDCP1 expression in bladder cancer patient tumor microarrays (TMAs). Left panel, representative images of METTL3 and CDCP1 IHC staining in tumor and paratumor of TMAs. Right panel, histogram of METTL3, CDCP1 expression in tumor and paratumor of TMAs was shown in the right panel. **b** Immunohistochemical staining with METTL3, CDCP1 antibodies in human cystitis, and bladder cancer tissues. Left panel, representative images of METTL3 and CDCP1 IHC staining in cystitis and bladder cancer tissues. Right panel, histogram of METTL3, CDCP1 expression in cystitis, and bladder cancer tissues. **c** MTS assay of cellular proliferation in KO-METTL3, KO-CDCP1 Cd-SV-HUC-1 cells. **d**, **e** Knockout of METTL3, CDCP1 inhibits cells migration (**d**) and invasion (**e**) in Cd-SV-HUC-1 cells. **f**, **g** Forced expression of CDCP1 partially rescues effects of METTL3 depletion on cells migration (**f**) and invasion (**g**) in Cd-SV-HUC-1. **h** Representation picture of tumor formation of xenograft in nude mice. **i** Summery of tumor volume of mice, which were measured every 3 days. **j** Weights of tumors in two groups were measured at the end point. **k** Representative images of METTL3 and CDCP1 IHC staining in tumors derived from METTL3-depleted and control T24 cells. Data are presented as ± SEM; *n* = 3. **p < 0.01, ***p < 0.001

Chemical carcinogens are important risk factors of bladder cancer. Our data identified METTL3-m^6^A-CDCP1 axis as a downstream target during chemical-induced transformation of uroepithelial cells; therefore, we evaluated the clinical relevance of METTL3-m^6^A-CDCP1 oncogenic signaling axis in bladder cancer with patient samples. Immunohistochemistry (IHC) staining of bladder cancer patient tumor microarray revealed that both METTL3 and CDCP1 are moderately or highly expressed in most of the bladder cancer samples, whereas their expression are weak or not detectable in the majority of the paratumor controls (Fig. 6a). We further investigated the association between METTL3-CDCP1 expression and bladder cancer progression. Determination of METTL3 and CDCP1 expression in human cystitis and bladder cancer tissues revealed that METTL3 and CDCP1 expressions are weak or not detectable in majority of the cystitis samples, although the METTL3 and CDCP1 are upregulated in the non-muscle-invasive bladder cancer samples. Moreover, the expression levels of METTL3 and CDCP1 are further elevated in the muscle-invasive bladder cancer samples (Fig. 6b). Taken together, METTL3 and CDCP1 expression are upregulated in human bladder cancer samples and the expression of METTL3 and CDCP1 are associated with bladder cancer progression.

### METTL3-m^6^A-CDCP1 axis is essential for the growth and progression of bladder cancer

The upregulated METTL3 and CDCP1 in bladder cancers and the correlation between high METTL3/CDCP1 expressions with advanced bladder cancer stages suggested the important function of METTL3-m^6^A-CDCP1 axis in regulation of bladder cancers. Therefore, we further studied the function of METTL3-m^6^A-CDCP1 axis in the growth and progression of chemical-transformed uroepithelial cells and bladder cancer cells. Our results revealed that depletion of METTL3 or CDCP1 modestly decreased proliferation (Fig. 6c) and significantly decreased the migration (Fig. 6d) and invasion (Fig. 6e) of the chemical-transformed Cd-SV-HUC-1 cells. Data from the bladder cancer T24 cells also supported the essential role of the METTL3-m^6^A-CDCP1 axis in regulation of proliferation, migration, and invasion of bladder cancers (Figure S8A–S8C). In addition, forced expression of CDCP1 partially rescued the effects of METTL3 depletion on migration (Fig. 6f) and invasion (Fig. 6g) in chemical-transformed Cd-SV-HUC-1 cells, further confirming CDCP1 as an essential downstream target of METTL3 in chemical carcinogenesis. Next, we studied the role of METTL3-m^6^A-CDCP1 axis in regulation of in vivo tumorigenesis using xenograft tumor models. Our data revealed that METTL3 depletion in T24 cells modestly reduced the bladder cancer growth in vivo (Fig. 6h–j). IHC analysis showed that the METTL3-depleted tumors had reduced CDCP1 expression (Fig. 6k). Overall, our in vitro and in vivo loss-of-function studies confirmed that the METTL3-m^6^A-CDCP1 axis is essential for the growth and progression of bladder cancer.

### METTL3-m^6^A-CDCP1 axis promotes uroepithelial cells transformation and tumorigenesis

**Fig. 7:**
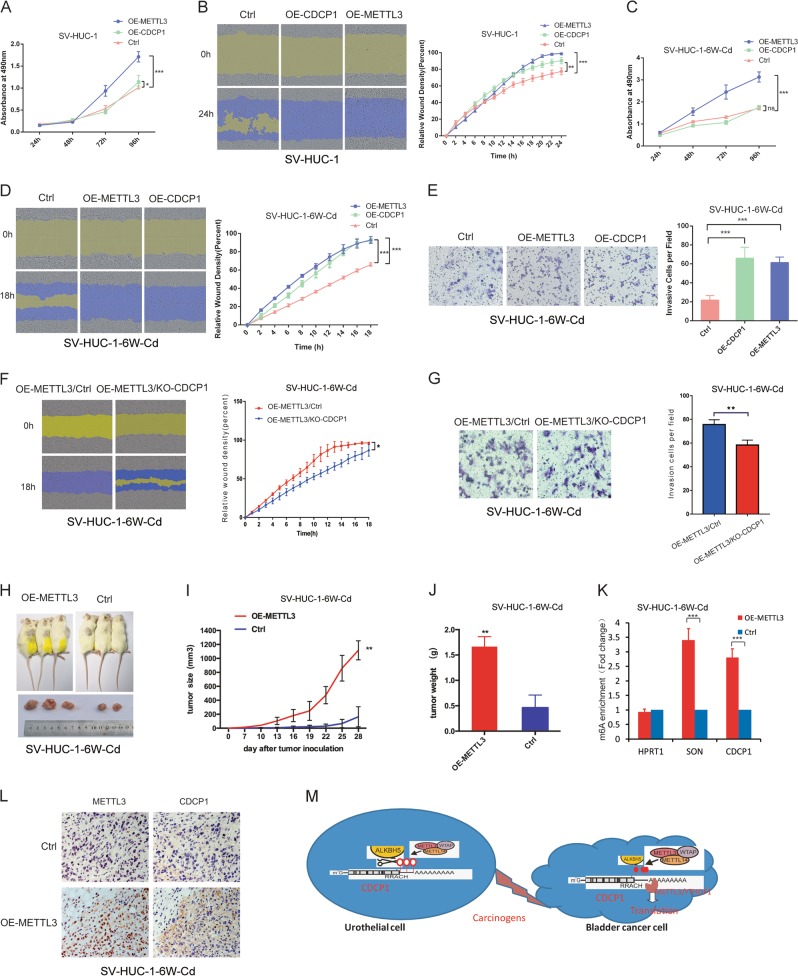
METTL3-mediated CDCP1 expression promotes uroepithelial cells transformation and tumorigenesis. **a** MTS assay of cellular proliferation in METTL3, CDCP1-overexpressing SV-HUC-1 cells (OE-METTL3 cells, OE-CDCP1 SV-HUC-1 cells). **b** METTL3, CDCP1 overexpression promotes cells migration in SV-HUC-1 cells. **c** MTS assay of cellular proliferation in Cd-induced OE-METTL3 SV-HUC-1 cells for 6 weeks (OE-METTL3 SV-HUC-1-6W-Cd) and Cd-induced OE-CDCP1 SV-HUC-1- cells for 6 weeks (OE-CDCP1 SV-HUC-1 -6W-Cd). **d**, **e** METTL3, CDCP1 overexpression enhances Cd-induced SV-HUC-1 cell migration (**d**) and invasion (**e**). **f**, **g** Depletion of CDCP1 partially reverses effects of METTL3 on migration (**f**) and invasion (**g**) in OE-METTL3 SV-HUC-1-6W-Cd cells. **h** Representation picture of tumor formation of xenograft in nude mice. **i** Summary of tumor volume of mice which were measured every 3 days. **j** Weights of tumors in two groups were measured at the end point. **k** m^6^A enrichment of CDCP1 mRNA 3′-UTR in the METTL3-overexpressing tumors. SON, positive control; HPRT1, negative control. **l** Representative images of METTL3 and CDCP1 IHC staining in subcutaneous tumor of SV-HUC-1-METTL3-6W-Cd and control cells. **m** Working model of m^6^A-mediated CDCP1 in chemical-induced bladder cancer development and metastasis

We next performed gain-of-function assays to study the role of METTL3-m^6^A-CDCP1 axis in uroepithelial transformation and bladder cancer tumorigenesis. As shown in Fig. 7a, overexpression of METTL3 significantly increased the proliferation of SV-HUC-1 uroepithelial cells. In addition, both METTL3 and CDCP1 can promote the migration of SV-HUC-1 cells (Fig. 7b). However, although METTL3-m^6^A-CDCP1 axis can promote the proliferation and migration of SV-HUC-1 uroepithelial cells in vitro, injection of METTL3 or CDCP1-overexpressing SV-HUC-1 cells into immunodeficient mice did not induce tumor formation in vivo (data not shown). Therefore, we further studied whether METTL3-m^6^A-CDCP1 axis and chemical carcinogen treatment have synergistic effect in promoting cells malignant transformation. For this purpose, we treated the METTL3- or CDCP1-overexpressing and the control SV-HUC-1 cells with chemical carcinogen Cd for 6 weeks. Our data showed the Cd treatment itself promotes the proliferation (Fig. 7c) and migration (Fig. 7d) of uroepithelial SV-HUC-1 cells. Overexpression of METTL3 together with Cd treatment can further promote the proliferation of the SV-HUC-1 cells (Fig. 7c). In addition, forced expression of METTL3 or CDCP1 functionally interplay with Cd treatment to promote the migration (Fig. 7d) and invasion (Fig. 7e) of SV-HUC-1 uroepithelial cells. Moreover, depletion of CDCP1 partially reverse the effects of METTL3 on migration (Fig. 7f) and invasion (Fig. 7g) in SV-HUC-1 cells treated with Cd for 6 weeks, suggesting CDCP1 is a major contributor to the function of METTL3 in cell transformation. These data further confirmed essential function of the METTL3-m^6^A-CDCP1 axis in promoting the chemical carcinogen-induced malignant transformation.

To study the interplay between METTL3 and chemical carcinogen in tumorigenesis in vivo, the Cd-treated METTL3-overexpressing and control SV-HUC-1 were implanted into immunodeficient mice by subcutaneous injection. Our data revealed that forced expression of METTL3 further promoted the Cd-induced carcinogenesis in the mouse xenograft model (Fig. 7h–j); consistent with previous results, the CDCP1 expression is upregulated in the METTL3-overexpressing tumors (Fig. 7l). Importantly, the m^6^A modification in CDCP1 mRNA also increased in the METTL3-overexpressing tumors (Fig. 7k). These data revealed the synergistic effect of METTL3 overexpression and chemical carcinogen in promoting oncogenic transformation and in vivo carcinogenesis. Overall, our data support the oncogenic function of METTL3-m^6^A-CDCP1 axis in promoting the in vitro and in vivo bladder cancer progression.

## Discussion

Chemical carcinogens induce a variety of genetic and epigenetic changes to promote oncogenic transformation in vitro and in vivo [27, 32, 33]. Aberrant DNA methylation is an important hallmark of cancer [34, 35]; chemical carcinogen treatment of normal epithelial cells causes abnormal promoter DNA methylations that are commonly seen in cancers and results in activation of key oncogenic signaling in tumorigenic process [32, 36]. Similar to DNA methylation, m^6^A modification of mRNA is catalyzed by methyltransferases to transfer methyl group from *S*-adenosyl-methionine to target sites; moreover, aberrant m^6^A modification is frequently found in cancers and is associated with cancer progression. However, the role of chemical treatment in m^6^A mRNA modification during chemical carcinogenesis is still unknown.

Indeed, more than 20 years ago, it was found that the increase in m^6^A mRNA methyltransferase activity was concomitant with adenovirus-induced rat embryo cells transformation [37]. However, the exact role of increased methyltransferase activity in the regulation of specific gene expression in transformed cells is largely unclear. In this study, we first profiled the m^6^A epitranscriptomic modifications in the chemical carcinogen-induced cellular transformation models. Similar to those of previous studies [38, 39], our results showed that the control and transformed cells transcriptomes contain 13,623~16,239 putative m^6^A sites within 7569~9039 coding gene transcripts (Table S3). Moreover, we not only found a subset of carcinogene-dependent or cell type-specific, dynamically altered m^6^A peaks but identified the common differential m^6^A peaks by comparing the methylation profiles of each set of control with the corresponding transformed cells (Fig. 1d, e, Table S4). These results suggested the complexity of m^6^A regulation mechanism of specific cell malignant transformation induced by different carcinogens. More importantly, our data uncovered the commonly dynamic m^6^A modification changes at the mRNAs of oncogenes such as CDCP1 during chemical carcinogenesis (Fig. 1).

CDCP1 is a transmembrame glycoprotein that is highly overexpressed in multiple cancer types and overexpression of CDCP1 is associated with poor prognosis in cancer patients [40–42]. CDCP1 interplays with the oncogenic Ras/ERK signaling to promote the cancer cell growth, migration, and invasion [41–43], and represents as a promising target for cancer therapeutics. Here we found that CDCP1 is posttranscriptionally regulated by the m^6^A pathway upon chemical carcinogenesis. Mechanistically, the m^6^A methyltransferase METTL3 and demethylase ALKBH5 mediate the dynamic m^6^A modification on CDCP1 mRNA. Furthermore, interestingly, METTL3 and YTHDF1, but not YTHDF2 and YTHDF3, bind to the 3′-UTR of CDCP1 mRNA and promote the CDCP1 translation. Our data support the novel function of the METTL3-YTHDF1 axis in promoting the m^6^A modification and translation of oncogenes such as CDCP1 upon chemical treatment, which could cooperate with other chemical-induced genetic and epigenetic changes to facilitate oncogenic transformation in chemical carcinogenesis (Fig. 7m).

The m^6^A mRNA methyltransferase METTL3 is upregulated in multiple types of cancers and can promote the m^6^A modification and expression of oncogenes including EGFR, TAZ, MYC, and SOX2 [18, 20, 24]. The increased m^6^A modification and translation of oncogene CDCP1 in the transformed uroepithelial cells indicates the potential role of METTL3/m^6^A/CDCP1 axis in bladder cancer oncogenesis. Analysis of METTL3 and CDCP1 expression in primary patient samples revealed that METTL3 and CDCP1 are strongly upregulated in the bladder cancer patient samples. Most importantly, the expression of METTL3 and CDCP1 is correlated with the progression status of the bladder cancers, further supporting the role of METTL3-mediated m^6^A pathway in bladder cancer oncogenesis.

Our in vitro and in vivo data revealed that inhibition of the METTL3-m^6^A-CDCP1 axis resulted in the decreased proliferation, migration, and invasion of bladder cancer cells and the chemical-transformed uroepithelial cells, suggesting that the METTL3-m^6^A-CDCP1 axis is essential for the growth and maintenance of bladder cancer cells and transformed cells. The fact that METTL3 and CDCP1 can both promote SV-HUC-1 migration and invasion, but only METTL3 can promote proliferation of SV-HUC-1 cells, suggests that METTL3 could have additional targets. Indeed, our m^6^A MeRIP-seq identified a subset of genes undergo m^6^A modification changes during chemical carcinogenesis and our data confirmed that CDCP1 is one of the key downstream target genes of METTL3 mainly related to migration and invasion. We further tested the function of METTL3-m^6^A-CDCP1 axis in bladder cancer initiation and found that forced expression of METTL3 or CDCP1 functionally interplay with Cd to promote the bladder cancer tumorigenesis, supporting the synergistic effect of METTL3-m^6^A-CDCP1 axis and chemical carcinogens in promoting oncogenic transformation.

## Conclusions

Overall, our study uncovered that altered m^6^A modifications as a novel epitranscriptomic mechanism in chemical carcinogenesis and suggested that METTL3-m^6^A-CDCP1 axis could be a promising therapeutic target for the treatment of chemical-induced cancers.

## Materials and methods

### Clinical samples

In this study, Formalin-fixed paraffin embedded (FFPE) tissues of 114 bladder cancer and 30 cystitis patients, who underwent radical cystectomy and bladder biopsies between February 2010 and October 2016, were obtained from the archives of the Department of Pathology of the First Affiliated Hospital of Sun Yat-sen University (Guangzhou, China) and the First Affiliated Hospital of Guangzhou Medical University (Guangzhou, China). Bladder cancer tissue microarray (HBlaU060CS01) were purchased from Shanghai Outdo Biotech.

### Cell culture and cell transformation

Human uroepithelial cells (SV-HUC-1) and 3-methylcholanthrene-transformed SV-HUC-1 cells (MC-SV-HUC T2) were obtained from the American Type Culture Collection (Manassas, VA). Human prostatic epithelial cell line RWPE-1 and human bladder cancer cell lines T24, UM-UC-3 was purchased from the Institute of Cell Biology, Chinese Academy of Sciences (Shanghai, China). Human bronchial epithelial cells (16HBE) and NiS-transformed cell lines (NSTC2) were generated as described previously [29]. RWPE-1 cells were cultured in KSF-M (Fisher Scientific) containing 50 mg/ml bovine pituitary extract and 5 ng/ml epidermal growth factor. SV-HUC-1 cells were cultured in F12K, T24 cells were cultured in RPMI 1640, and UM-UC-3, 16HBE, NSTC2 cells were cultured in Minimum Eagle’s medium (MEM). All medium were supplemented with 10% fetal bovine serum (FBS; Gibco, USA), in a humidified air atmosphere of 5% CO_2_ at 37 °C. All the cell lines were tested negative for mycoplasma contamination using a PCR-based universal mycoplasma detection kit. CdCl_2_-induced cells transformation assay were performed as described previously [44, 45]. Briefly, SV-HUC-1 and RWPE-1 cells were maintained continuously in medium containing 10 µM CdCl_2_ for 8 and 10 weeks, respectively, and then the cells were cultured in Cd-free medium for 2 weeks.

### Tumorigenicity in NOD/SCID mice

To test for malignant transformation, 1 × 10^7^ cells were inoculated subcutaneously in the dorsal thoracic midline of ten NOD/SCID mice (Weitong Lihua Experimental Animal Technology Co. Ltd). Tumor formation and growth were assessed every 3 days. Tumor volume = Length × Width^2^ × 0.52. Tumor samples were paraffin-embedded, sectioned, stained with hematoxylin and eosin, and analyzed by light microscopy.

### m^6^A methylated RNA immunoprecipitation sequencing

MeRIP-seq and data analysis were performed as described previously [46]. Briefly, total RNA was extracted from cells using Trizol (Invitrogen) following the manufacturer’s instructions. mRNA was first purified from total RNA using Gen Elute mRNA Miniprep Kit (Sigma-Aldrich). Then 5 μg mRNA was fragmented using a RNA fragmentation kit (Ambion) and immunoprecipitated with the mixture of Protein A beads (Thermo Fisher) and anti-m^6^A antibody (Synaptic Systems); the immunoprecipitated RNA was washed and eluted by competition with m^6^A nucleotide solution (Sigma-Aldrich). The purified RNA fragments from m^6^A MeRIP were used for library construction and sequenced with Illumina Nextseq500. Reads mapping, m^6^A peak calling, motif search, and differentially methylated peaks were analyzed by exomePeak R/Bioconductor package as described [47].

### RNA-binding protein immmunoprecipitation

The RNA samples was isolated from HUVEC primary cells and cDNA synthesized by Reverse Transcription PCR using TransScript® All-in-One First-Strand cDNA Synthesis SuperMix for quantitative PCR (qPCR) (Transgene). The YTHDF1, YTHDF2, YTHDF3, and METTL3 CDS regions were PCR amplified and cloned into pcDNA3-FLAG2AB using HindIII and EcoRI restriction sites. The 2AB-FLAG-YTHDF (3.5 μg) and PLEX-CDCP1 (4 μg) plasmids were co-transfected into 10^7^ METTL3-overexpressing 293T cells by Lipofectamine 3000. The medium was replaced with fresh Dulbecco’s modified Eagle’s medium (DMEM)/10% FBS 6–8 h after transfection. Forty-eight hours later, cell lysate was using the Magna RIP™RNA-Binding Protein Immunoprecipitation Kit (Millipore). The lysate was then immunoprecipitated with 50 μl protein A/G magnetic beads and 5 μg anti-FLAG antibody(Sigma) for 30 min at room temperature. Normal Rabbit IgG was used as a negative control. The magnetic bead-bound complexes were immobilized with magnet and washed with RIP Wash Buffer. The precipitated RNA samples were extracted with Phenol:chloroform purification, then analyzed by qPCR.

### Dual-luciferase reporter assay

Luciferase reporter assay was performed with the Dual-Luciferase Reporter Assay System (Promega) according to the manufacturer’s descriptions. Cells were seeded into 24-well plates (about 3 × 10^5^ cells/well) 1 day before the transfection. After 24 h, the cells were co-transfected with the psiCHECK^TM^-2 Vector (Promega), which is used for internal normalization and various constructs containing the seed sequence or mutant seed sequence of CDCP1 mRNA 3′-UTR. The relative luciferase activities were accessed 48 h post transfection by SYNERGY microplate reader (BioTek). Each group was performed at least three times.

### In vitro transcription and translation

The psiCHECK^TM^-2 vectors with WT or mutant m^6^A motifs of CDCP1 mRNA 3′-UTR were treated with Acc651 and Mlu1 to linearize the DNA templates. The in vitro transcription was performed with mMESSAGE mMACHINE Kit (Ambion) according to the manufacturer’s instructions. The transcribed mRNA samples were purified with Phenol:chloroform extraction and isopropanol precipitation. The in vitro translation of the purified mRNA samples was performed using Flexi Rabbit Reticulocyte Lysate System (Promega), then the reaction was tested for the synthesis of functional luciferase using the standard luciferase assay.

### Immunofluorescence and immunohistochemistry

The cells were fixed with 4% formaldehyde for 15 min and then blocked with 0.1% Triton X-100 in phosphate-buffered saline (PBS) for 5 min and with 1% bovine serum albumin (BSA) for 60 min at room temperature. Immunofluorescence staining was incubated with appropriate primary antibodies and then performed with appropriate Alexa Fluor 488 and Alexa Fluor 568 secondary antibodies (dilution 1:1000) for 60 min at room temperature, respectively. Nuclei were counterstained with 4′,6-diamidino-2-phenylindole. Images were taken with a ZEISS Axio Imager Z2 Upright Microscope.

Paraffin sections are baked for 2 h at 55 °C. Before immunostaining, paraffin sections were deparaffinized, rehydrated through an alcohol series followed by antigen retrieval with sodium citrate buffer. The sections were then treated in 3% hydrogen peroxide for 30 min and subsequently blocked with 5% normal goat serum. After blocking, the sections were incubated with appropriate primary antibodies at 4 °C overnight. IHC staining was performed with horseradish peroxidase (HRP) conjugates using Diaminobenzidine (DAB) detection. Images were taken with a ZEISS Axio Imager.Z2 Microscope.

### RNA isolation and quantitative real-time PCR

RNA was isolated using TRIzol (Life Technologies) following the manufacturer’s protocol. cDNA was generated using the transScript All-in-One First-Strand cDNA Synthesis SuperMix for qPCR (Transgen). Quantitative real-time PCR using Fast SYBR Green PCR Master Mix (Applied Biosystems) was performed on a Step-One Fast Real-time PCR System (Applied Biosystems). For RNA stability assay, the cells were plated in a 6 cm dish and incubated with actinomycin D (Santa Cruz) at 5 mg/ml for indicated time points. Total RNA samples were then isolated for qPCR analysis. The quantitative PCR primers were listed in Table S1.

### Sucrose gradient centrifugation and polysomal fractionation

Polysomal fractionation to categorize translationally active transcripts was performed using sucrose gradient centrifugation as previously described [48]. Cell lines for polysomal fractionation were first lysed using Polysome cell extraction buffer (50 mM MOPS, 15 mM MgCl_2_, 150 mM NaCl, 100 μg/ml CHX, 0.5% Triton X-100, 1 mg/ml Heparin, 200 U RNaseout, 2 mM phenylmethylsulfonyl fluoride (PMSF) 1 μM Benzamine). Following cell lysis, nuclei and cell debris were cleared by centrifugation at 13,000 × *g* for 10 min at 4 °C. One milliliter of supernatants was laid on the top of 11 ml 10~50% sucrose gradient tube, then centrifuged at 36,000 r.p.m. for 2 h 30 min at 4 °C with max break (Beckman coulter SW 41 Ti rotor) at 4 °C. Then the RNA in polysome fraction was extracted and subjected to real-time PCR.

### Immunoblotting (western blotting)

Cells were washed twice with ice-cold PBS and ruptured with RIPA buffer (Sigma-Aldrich) containing 5 mM EDTA, PMSF, cocktail inhibitor, and phosphatase inhibitor cocktail. Cell extracts were centrifuged for 20 min at 10,000 × *g* and supernatants were then collected. Cell lysates were resolved by SDS-polyacrylamide gel electrophoresis and transferred onto polyvinylidene difluoride membranes. Membranes were blocked for 1 h with 5% BSA (Sigma-Aldrich) in Tris-buffered saline containing 0.1% Tween 20 and incubated overnight at 4 °C with anti-METTL3 antibody (Proteintech), anti-ALKBH5 antibody (Sigma-Aldrich), anti-FTO (PhosphoSolutions), anti-β-Actin (Cell Signal Technology), anti-GAPDH (Cell Signal Technology), anti-FLAG (Sigma-Aldrich), and anti-YTHDF1, anti-YTHDF2, and anti-YTHDF3 (Proteintech). Membranes were washed for 30 min with Tris-buffered saline containing 0.1% Tween 20, incubated for 1 h with appropriate secondary antibodies conjugated to HRP, and developed using chemiluminescent substrates.

### Plasmids and mutagenesis assays

METTL3, ALKBH5, FTO, and CDCP1 expression plasmids were generated by cloning the full-length Open Reading Frame (ORF) of human METTL3 gene (NM_019852), ALKBH5 (NM_017758), FTO(NM_001080432), and CDCP1(NM_022842) into LentiORF PLEX vector. YTHDF1(NM_017798), YTHDF2(NM_001172828), YTHDF3(NM_001277813), WT, and mutant of METTL3 were cloned to pcDNA3-Flag2AB vector. 3′-UTR of CDCP1 were cloned from HUVEC cells into psiCHECK™-2 vector (Promega). The psiCHECKTM-2-CDCP1 3′-UTR containing the m^6^A mutant motifs (A was replaced by T) were site-directed mutagenesis. Transfections were performed using Lipofectamine® 3000 Transfection kit for plasmid and Lipofectamine® RNAiMAX Reagent (Thermo Fisher Scientific) for small interfering RNA following the manufacturer’s protocols. The cloning and mutagenesis primers are listed in Table S1.

### CRISPR-mediated stable knockout cell lines

Lentiviral vectors expressing single guide RNAs (sgRNAs) targeting METTL3, ALKBH5, FTO, or CDCP1 were generated according to the lentiCRISPRv2 protocol. lentiCRISPRv2 was a gift from Feng Zhang [49] (Addgene plasmid # 52961). The sgRNAs with highest scores and least off-target effects were chosen using the online tool (https://chopchop.rc.fas.harvard.edu/index.php). The sequences of sgRNAs were listed in Table S1. The lentiviral vectors were co-transfected with packaging vectors psPAX2 and VSVG (Addgene) into 293T cells for lentivirus production. To establish stable cell lines, the cells were transduced using the above lentiviruses with polybrene (8 mg/ml, Sigma). Seventy-two hours after transduction, cells were selected with 1 μg/ml puromycin for 5–7 days.

### Proliferation assay

Cells (5 × 10^3^) were seeded into 96-well plates and incubated the plates at 37 °C in a humidified 5% CO_2_ atmosphere. Cellular proliferation was measured with CellTiter 96® AQueous One Solution Cell Proliferation Assay (MTS, 3-(4,5-dimethylthiazol-2-yl)-5-(3-carboxymethoxyphenyl)-2-(4-sulfophenyl)-2H-tetrazolium) (Promega). Briefly, 24, 48, 72, and 96 h later, 20 μl/well MTS solution was added and then the cells were incubated at 37 °C for 2 h. The absorbance at 490 nm value was recorded by the SYNERGY microplate reader (BioTEK).

### Migration assay

Cell migration assay was performed with the IncuCyte™ 96-well Real-Time Cell Migration System (Essen Bioscience). Before initiating a 96-well assay, cells (1 × 10^5^) were grown to confluence in a 96-well Essen ImageLock™ plate in a standard CO_2_ incubator. The 96-pin WoundMaker simultaneously created precise and reproducible wounds in all wells of a 96-well ImageLock plate by gently removing the cells from the confluent monolayer using an array of 96 pins. After washing by cold PBS, the plate was placed inside the IncuCyte. The software was set to scan the experiment every hour for migration assays using “Scratch Wound” as the “Experiment Type”. The data were analyzed by the relative wound density and the image collection was created using the representative phase-contrast images. Additional statistics and graphing were completed using GraphPad Prism following data export.

### Transwell^TM^ invasion assay

Cell invasion assay was performed in a 24-well Transwell™ (Costar). The upper chamber surface of the filter was coated with Matrigel (Corning) before the experiment. The cells were prepared (1 × 10^5^/200 μl) with serum-free DMEM and loaded into the upper chamber. DMEM medium containing 20% FBS was added to the bottom chamber as the chemoattractant. After 24 h incubation, wet cotton was used to remove the non-invaded cells from the wells. The cells were fixed with carbinol for 15 min at room temperature, stained with 0.1% crystal violet for 20 min, and quantified by counting the total number of cells in four independent areas under the ZEISS Axio Imager.Z2 Microscope.

### Quantification and statistical analysis

Data are presented as the mean ± SEMs or SDs. Statistical analyses were performed in GraphPad Prism 6 (GraphPad Software, Inc.) using unpaired two-tailed Student’s *t*-test to compare differences between two groups with significance of *P* < 0.05. One-way analysis of variance with multiple comparisons tests was used to compare three or more groups with significance of *P* < 0.05.

## Supplementary information

Live cell imaging of scratch wound assay in Cd-SV-HUC-1-V2 cells

Live cell imaging of scratch wound assay in Cd-SV-HUC-1-KO-METTL3 cells

Live cell imaging of scratch wound assay in Cd-SV-HUC-1-KO-CDCP1 cells

Live cell imaging of scratch wound assay in SV-HUC-1-Con cells

Live cell imaging of scratch wound assay in SV-HUC-1-METTL3 cells

Fig.S1 CdCl2-induced SV-HUC-1 and RWPE-1 cells transformation model

Fig. S2 Depletion of METTL3 inhibits CDCP1 translation in T24 cells

Fig. S3 Son and HPRT1 mRNA stability in control, OE-METTL3 and KO-ALKBH5 SV-HUC-1 cells

Fig.S4 FTO couldn’t affect CDCP1 expression

Fig. S5 Relative luciferase activity of psiCHECK™-2

Fig.S6 Effect of immunoprecipitation with an anti-FLAG antibody in RIP experiment

Fig.S7 Identification of stable OE or KO-METTL3, CDCP1 cells

Fig.S8 Depletion of METTL3 and CDCP1 inhibit proliferation, migration and invasion in T24 cells

Tab.S1 Primers used in this study

Tab.S2 Reagent or Resource

Tab. S3 Number of peaks and genes in the control and transformed cells by MeRIP-Seq

Tab. S4 Number of differential peaks and genes in each set of control to the corresponding transformed cells

## Data Availability

MeRIP-seq data are deposited at the Gene Expression Omnibus database with the accession Number GSE112970.
