# Supplementary material for: Dynamic m6A mRNA methylation reveals the role of METTL3-m6A-CDCP1 signaling axis in chemical carcinogenesis
Source: Oncogene. 2019 Feb 22;38(24):4755–72. doi: 10.1038/s41388-019-0755-0 (PMC6756049; doi:10.1038/s41388-019-0755-0)
Supplement: Supplementary file 6 — Fig.S1 CdCl2-induced SV-HUC-1 and RWPE-1 cells transformation model [file 41388_2019_755_MOESM6_ESM.docx]

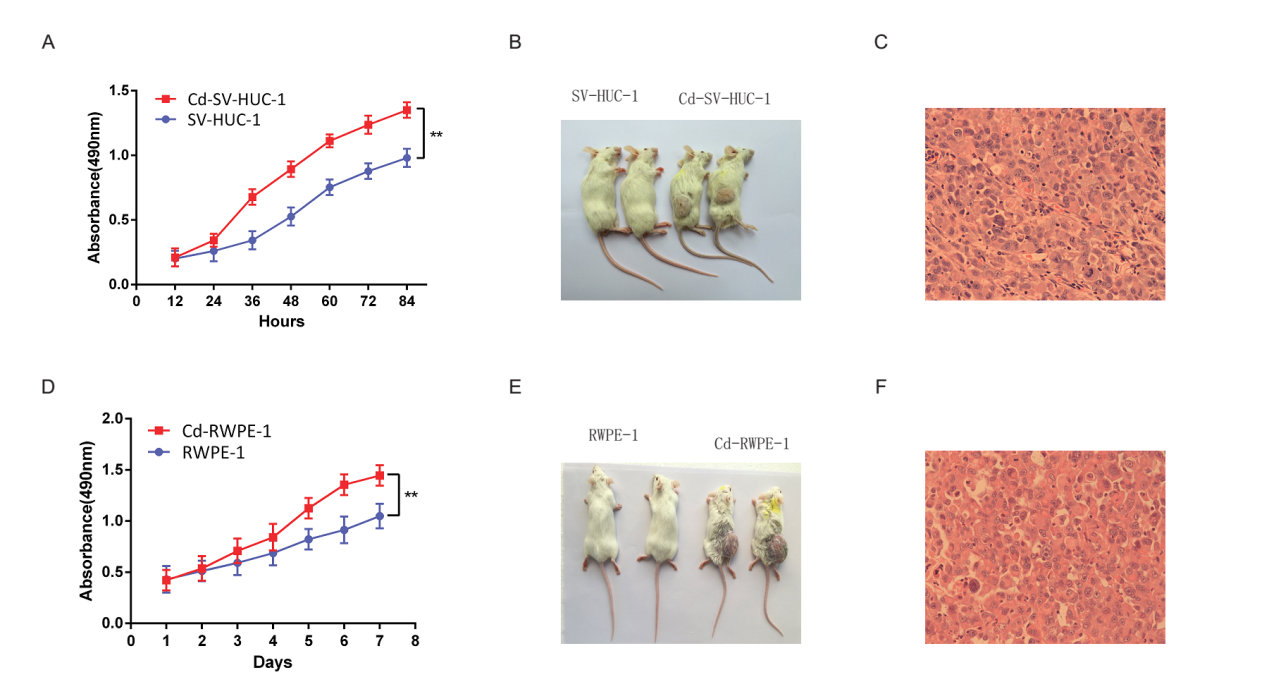


**Figure S1** **CdCl_2_-induced SV-HUC-1 and RWPE-1 cells transformation model**

A, B, C, CdCl_2_-induced SV-HUC-1 cells transformation. D, E, F, CdCl_2_-induced RWPE-1 cells transformation. A, D, MTS assay of cellular proliferation in the control and transformed cells. B, E, Subcutaneous tumor model of transformed cells. C, F, Representative images of H&E staining in tumor heterotransplants produced by the transformed cells
