# Supplementary material for: Dynamic m6A mRNA methylation reveals the role of METTL3-m6A-CDCP1 signaling axis in chemical carcinogenesis
Source: Oncogene. 2019 Feb 22;38(24):4755–72. doi: 10.1038/s41388-019-0755-0 (PMC6756049; doi:10.1038/s41388-019-0755-0)
Supplement: Supplementary file 7 — Fig. S2 Depletion of METTL3 inhibits CDCP1 translation in T24 cells [file 41388_2019_755_MOESM7_ESM.docx]

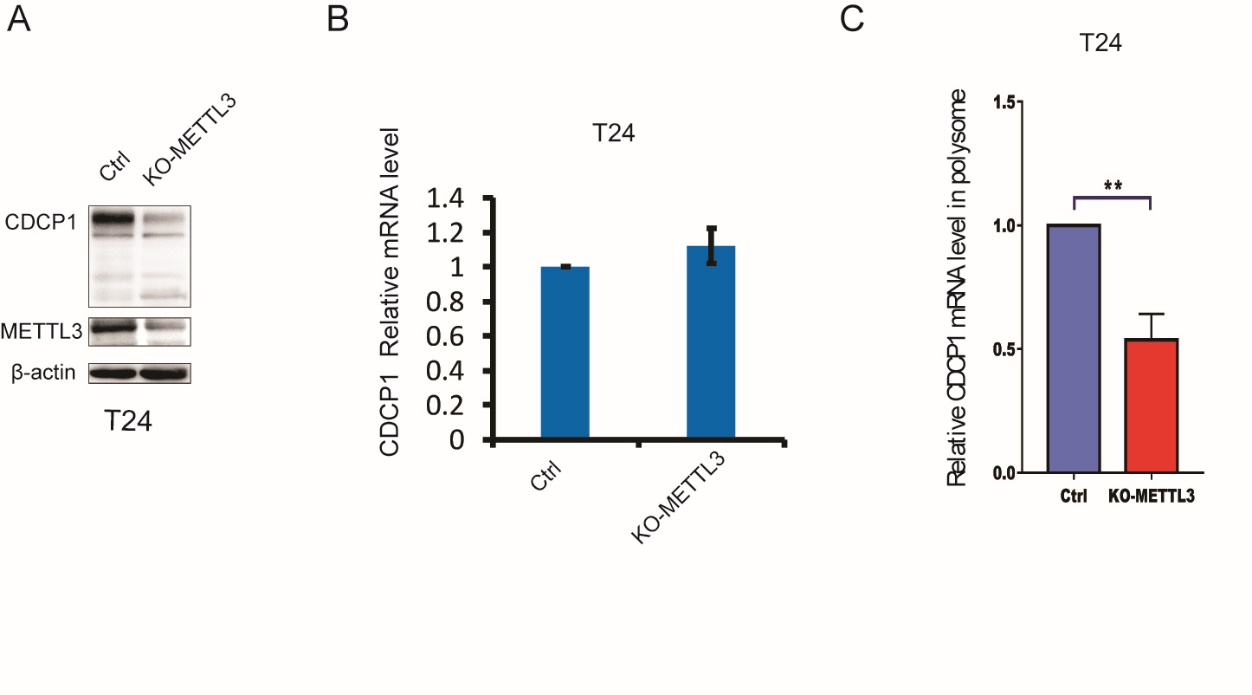


**Figure S2 Depletion of METTL3 inhibits CDCP1 translation in T24 cells**

A, Western blotting of CDCP1 in control and METTL3-depleted T24 cells. B, qRT-PCR analysis of CDCP1 mRNA expression in control and METTL3-depleted T24 cells. C, CDCP1 mRNA expression of polysomal fractionated RNA in control and METTL3-depleted T24 cells. All bar plot data are means ± SEM of three independent experiments. **p < 0.01, ***p < 0.001.
