# Supplementary material for: Dynamic m6A mRNA methylation reveals the role of METTL3-m6A-CDCP1 signaling axis in chemical carcinogenesis
Source: Oncogene. 2019 Feb 22;38(24):4755–72. doi: 10.1038/s41388-019-0755-0 (PMC6756049; doi:10.1038/s41388-019-0755-0)
Supplement: Supplementary file 8 — Fig. S3 Son and HPRT1 mRNA stability in control, OE-METTL3 and KO-ALKBH5 SV-HUC-1 cells [file 41388_2019_755_MOESM8_ESM.docx]

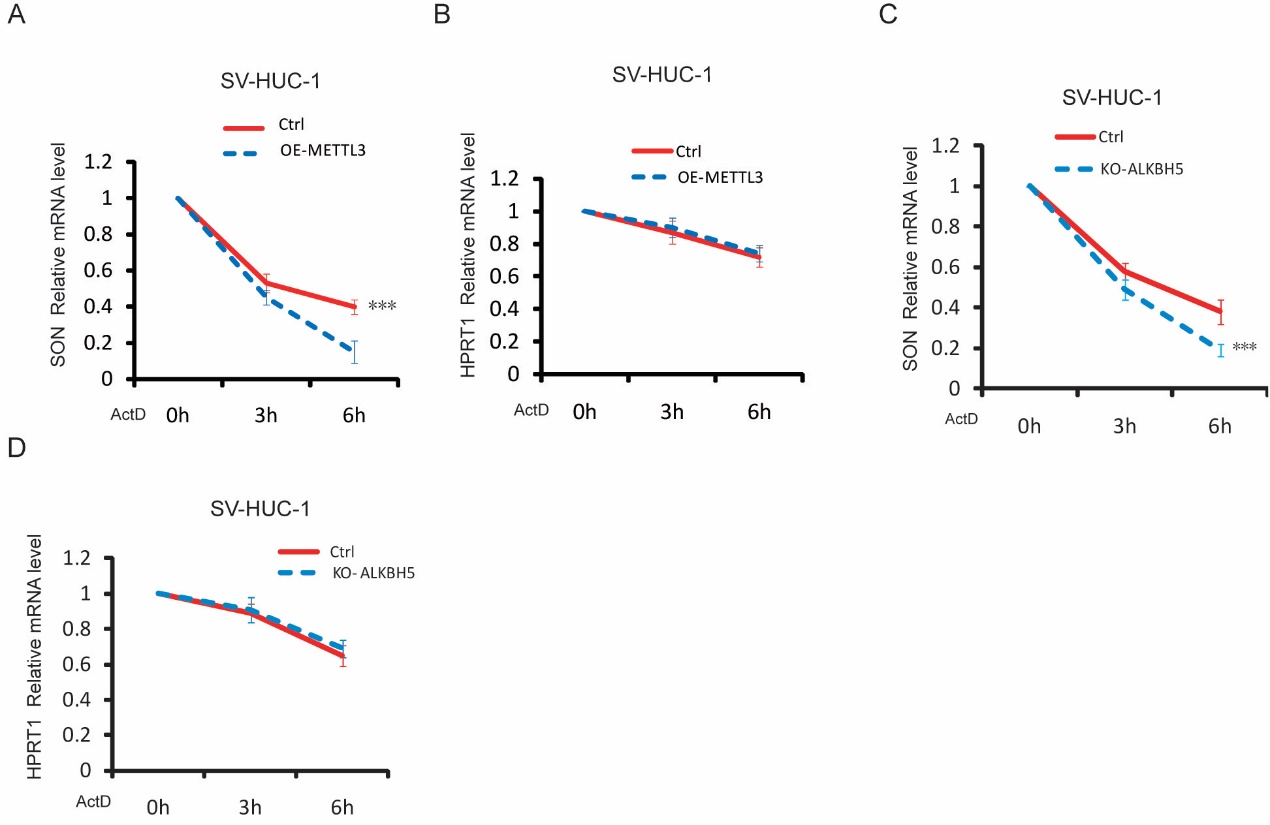


**Figure S3 Son and HPRT1 mRNA stability in control, OE-METTL3 and KO-ALKBH5 SV-HUC-1 cells.**

A, Son (positive control) mRNA stability in control and OE-METTL3 SV-HUC-1 cells. B, HPRT1 (negative control) mRNA stability in control and OE-METTL3 SV-HUC-1 cells. C, Son mRNA stability in control and KO-ALKBH5 SV-HUC-1 cells. D, HPRT1 mRNA stability in control and KO-ALKBH5 SV-HUC-1 cells.
