# Supplementary material for: Dynamic m6A mRNA methylation reveals the role of METTL3-m6A-CDCP1 signaling axis in chemical carcinogenesis
Source: Oncogene. 2019 Feb 22;38(24):4755–72. doi: 10.1038/s41388-019-0755-0 (PMC6756049; doi:10.1038/s41388-019-0755-0)
Supplement: Supplementary file 9 — Fig.S4 FTO couldn’t affect CDCP1 expression [file 41388_2019_755_MOESM9_ESM.docx]

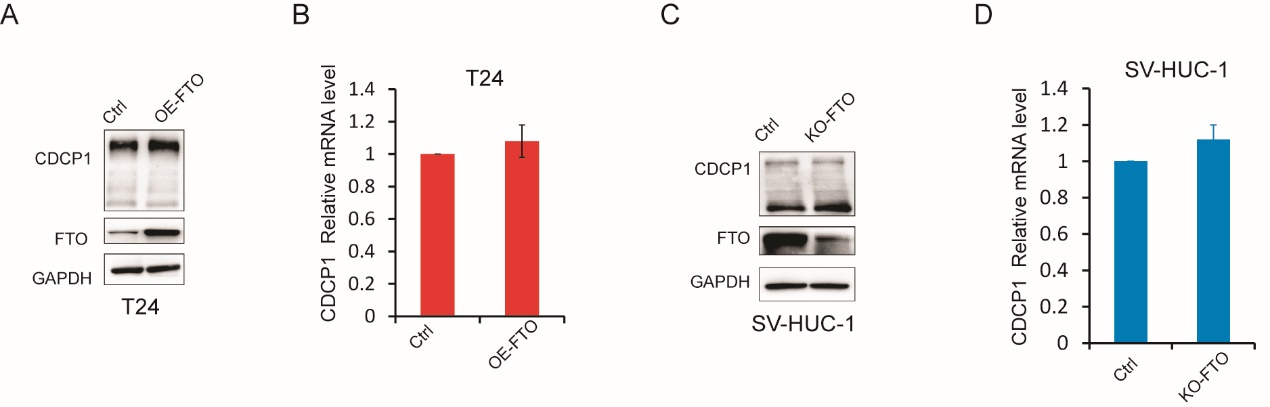


**Figure S4 FTO couldn’t affect CDCP1 expression**

A, Western blotting of CDCP1 in control and FTO-overexpressing T24 cells. B, qRT-PCR analysis of CDCP1 mRNA expression in control and FTO-overexpressing T24 cells. C, Western blotting of CDCP1 in control and FTO-depletion SV-HUC-1 cells. D, qRT-PCR analysis of CDCP1 mRNA expression in control and FTO-depletion SV-HUC-1 cells.
