# Supplementary material for: Dynamic m6A mRNA methylation reveals the role of METTL3-m6A-CDCP1 signaling axis in chemical carcinogenesis
Source: Oncogene. 2019 Feb 22;38(24):4755–72. doi: 10.1038/s41388-019-0755-0 (PMC6756049; doi:10.1038/s41388-019-0755-0)
Supplement: Supplementary file 10 — Fig. S5 Relative luciferase activity of psiCHECK™-2 [file 41388_2019_755_MOESM10_ESM.docx]

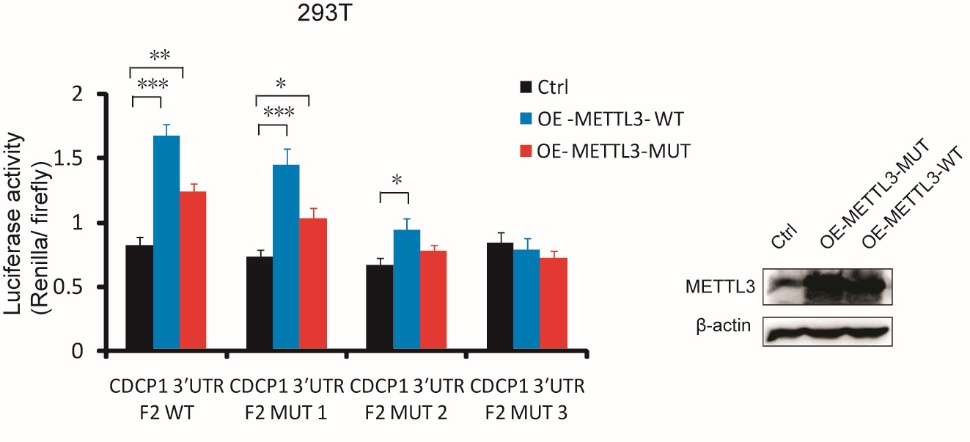


Figure S5 Relative luciferase activity of psiCHECK™-2- CDCP1 3′-UTR with either F2 wild-type (F2 WT) or 1,2,3 mutant m^6^A sites (F2 MUT1, F2 MUT2, F2 MUT3) in control and OE-METTL3-WT, OE-METTL3-MUT 293T cells.
