# Supplementary material for: Dynamic m6A mRNA methylation reveals the role of METTL3-m6A-CDCP1 signaling axis in chemical carcinogenesis
Source: Oncogene. 2019 Feb 22;38(24):4755–72. doi: 10.1038/s41388-019-0755-0 (PMC6756049; doi:10.1038/s41388-019-0755-0)
Supplement: Supplementary file 11 — Fig.S6 Effect of immunoprecipitation with an anti-FLAG antibody in RIP experiment [file 41388_2019_755_MOESM11_ESM.docx]

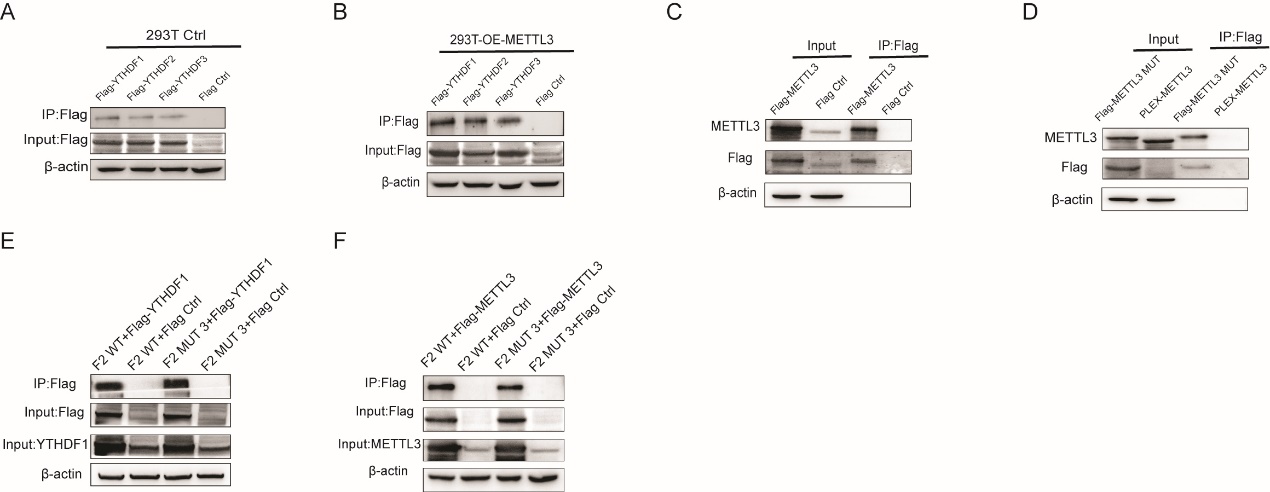


**Figure S6 Effect of immunoprecipitation with an anti-FLAG antibody in RIP experiment**

A, B, Western blotting of FLAG expression in Input and IP lysates from control(A) and METTL3-overexpressing 293T cells(B) transfected with FLAG-tagged YTHDF1, YTHDF2, YTHDF3 or control 2AB vectors. C, D, Western blotting of FLAG and METTL3 in Input and IP lysates from 293T cells transfected with FLAG-tagged METTL3-WT(C), METTL3-MUT(D), non-FLAG-tagged METTL3 or control 2AB vectors. E, Western blotting of FLAG and YTHDF1 in Input and IP lysates from METTL3-overexpressing 293T cells co-transfected with FLAG-tagged YTHDF1 vector and psiCHECK™-2- CDCP1 3′-UTR containing m^6^A sites (F2 WT) or mutant 3 m^6^A sites (F2 MUT3). F, Western blotting of FLAG and METTL3 in Input and IP lysates from 293T cells co-transfected with FLAG-tagged METTL3 vector and psiCHECK™-2- CDCP1 3′-UTR containing m^6^A sites (F2 WT) or mutant 3 m^6^A sites (F2 MUT3).
