# Supplementary material for: Dynamic m6A mRNA methylation reveals the role of METTL3-m6A-CDCP1 signaling axis in chemical carcinogenesis
Source: Oncogene. 2019 Feb 22;38(24):4755–72. doi: 10.1038/s41388-019-0755-0 (PMC6756049; doi:10.1038/s41388-019-0755-0)
Supplement: Supplementary file 12 — Fig.S7 Identification of stable OE or KO-METTL3, CDCP1 cells [file 41388_2019_755_MOESM12_ESM.docx]

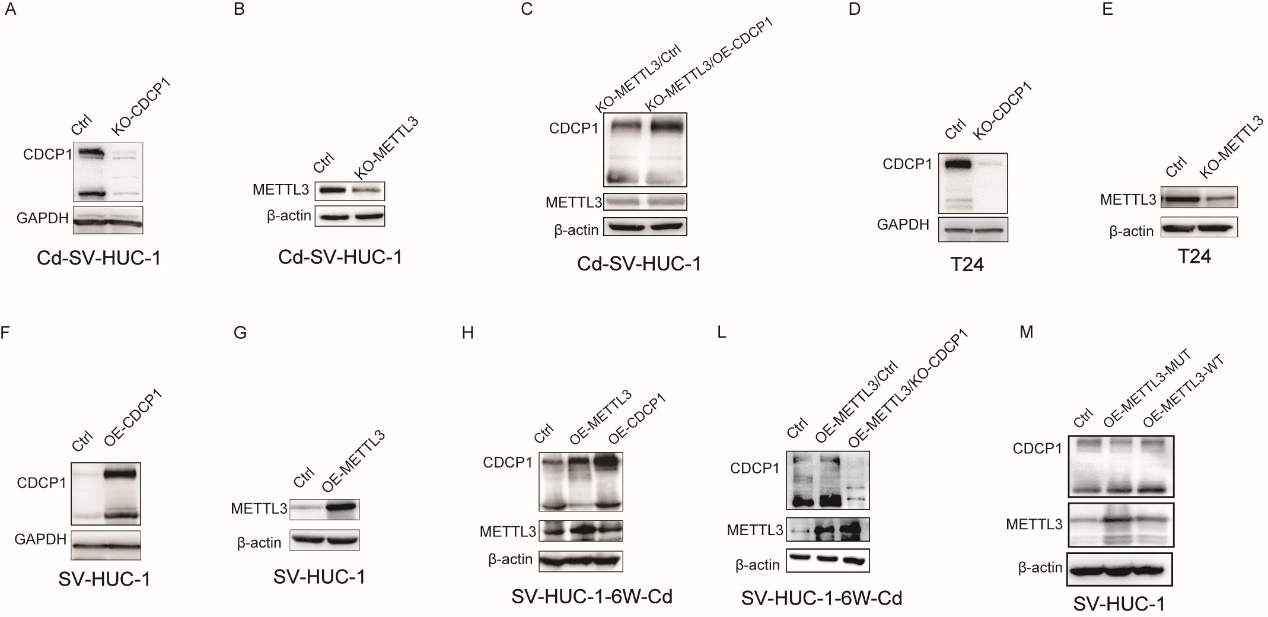


**Figure S7 Identification of stable OE or KO-METTL3, CDCP1 cells**

A, Western blotting of CDCP1 in control and CDCP1-depleted Cd-SV-HUC-1 cells. B, Western blotting of METTL3 in control and METTL3-depleted Cd-SV-HUC-1 cells. C, Western blotting of CDCP1 in METTL3-depleted Cd-SV-HUC-1 control cells and CDCP1-overexpressing METTL 3-depleted Cd-SV-HUC-1 cells. D, Western blotting of CDCP1 in control and CDCP1-depleted T24 cells. E, Western blotting of METTL3 in control and METTL3-depleted T24 cells. F, Western blotting of CDCP1 in control and CDCP1-overexpressing SV-HUC-1 cells. G, Western blotting of METTL3 in control and METTL3-overexpressing SV-HUC-1 cells. H, Western blotting of CDCP1 and METTL3 in Cd-induced control, Cd-induced CDCP1-overexpressing and Cd-induced METTL3-overexpressing SV-HUC-1 cells for 6 weeks. L, Western blotting of CDCP1 and METTL3 in Cd-induced control, Cd-induced METTL3-overexpressing control and Cd-induced CDCP1-depleted METTL3-overexpressing SV-HUC-1 cells for 6 weeks. M, Western blotting of CDCP1 and METTL3 in control, METTL3 WT-overexpressing and METTL3 MUT-overexpressing SV-HUC-1 cells.
