# Supplementary material for: Dynamic m6A mRNA methylation reveals the role of METTL3-m6A-CDCP1 signaling axis in chemical carcinogenesis
Source: Oncogene. 2019 Feb 22;38(24):4755–72. doi: 10.1038/s41388-019-0755-0 (PMC6756049; doi:10.1038/s41388-019-0755-0)
Supplement: Supplementary file 13 — Fig.S8 Depletion of METTL3 and CDCP1 inhibit proliferation, migration and invasion in T24 cells [file 41388_2019_755_MOESM13_ESM.docx]

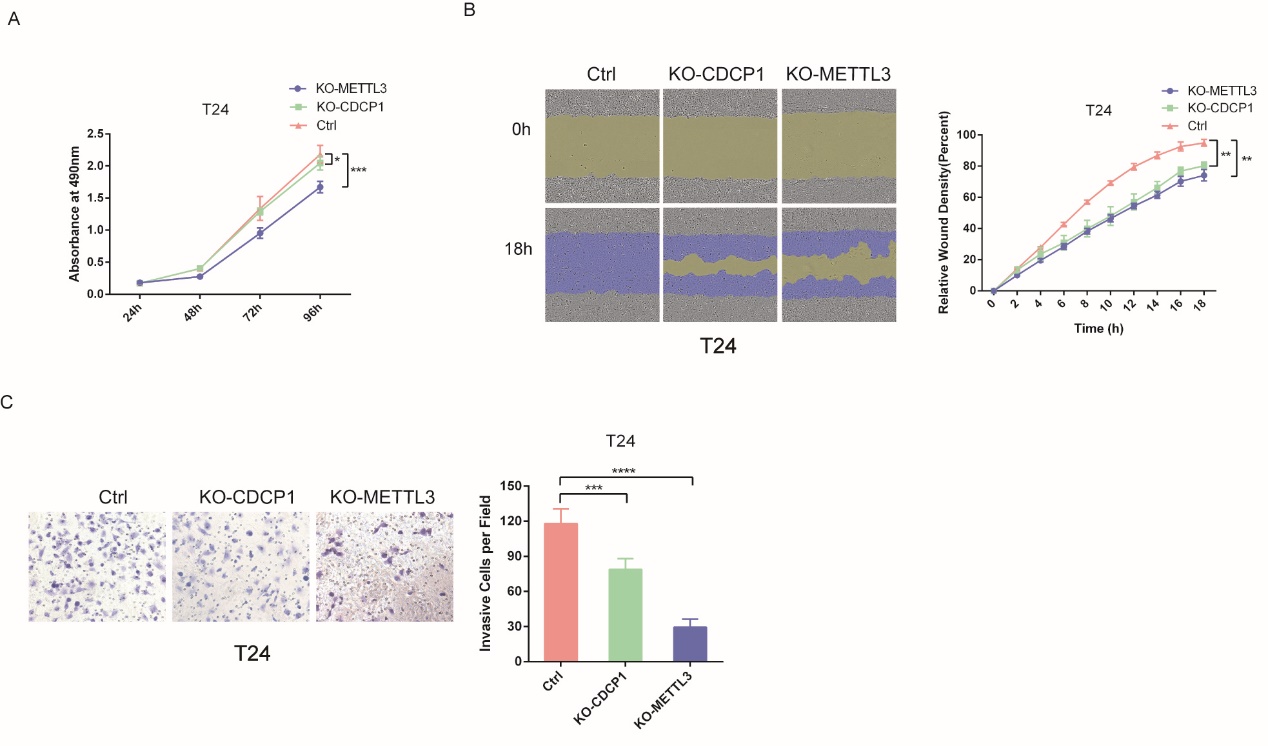


Figure S8 Depletion of METTL3 and CDCP1 inhibit proliferation, migration and invasion in T24 cells

A, MTS assay of cellular proliferation in KO-METTL3, KO-CDCP1 T24 cells. B, C, Knockout of METTL3, CDCP1 inhibits cells migration (B) and invasion(C) in T24 cells.
