# Supplementary material for: Dynamic m6A mRNA methylation reveals the role of METTL3-m6A-CDCP1 signaling axis in chemical carcinogenesis
Source: Oncogene. 2019 Feb 22;38(24):4755–72. doi: 10.1038/s41388-019-0755-0 (PMC6756049; doi:10.1038/s41388-019-0755-0)
Supplement: Supplementary file 14 — Tab.S1 Primers used in this study [file 41388_2019_755_MOESM14_ESM.docx]

**Table S1.**Primers used in this study

| **Primer Name** | **Primer sequence(5’——3’)** | |
| --- | --- | --- |
|  | **F** | **R** |
| Cloning Primers |  |  |
| METTL3-CDS | ACACTAGTGCCACCATGTCGGACACGTGGAGCTCTA *(Spe I)* | TCGCGGCCGCCTATAAATTCTTAGGTTTAGAGATGATACCATCTG (*Not I*) |
| ALKBH5-CDS | ACACTAGTGCCACCATGGCGGCCGCCAGCGGCTACA *(Spe I)* | TCCTCGAGTCAGTGCCGCCGCATCTTCACCTTTCGG *(Xho I)* |
| FTO-CDS | ACACTAGTGCCACCATGAAGCGCACCCCGACTGCCG *(Spe I)* | TCCTCGAGCTAGGGTTTTGCTTCCAGAAGCTGACCTCTG *(Xho I)* |
| CDCP1-CDS | ACACTAGTATGGCCGGCCTGAACTGCGGGGTCT *(Spe I)* | TCCTCGAGCACACAGCAAATGATGAGCCCGAGG *(Xho I)* |
| 2AB-YTHDF1-CDS | CCCAAGCTTATGTCGGCCACCAGCGTG *(Hind III)* | CCGGAATTCTCATTGTTTGTTTCGACTCTGCCGT *(EcoR I)* |
| 2AB-YTHDF2-CDS | CGGGATCCATGTCGGCCAGCAGCCTCTT *(BamH I)* | AAATATGCGGCCGCTTATTTCCCACGACCTTGACGTTCC (*Not I*) |
| 2AB-YTHDF3-CDS | CCAAGCTTGGATGTCAGCCACTAGCGTGGATCAGA *(Hind III)* | CGGAATTCCGTTATTGTTTGTTTCTATTTCTCTCC *(EcoR I)* |
| 2AB-METTL3-CDS | CCCAAGCTTATGTCGGACACGTGGAGCTCTA *(Hind III)* | AAATATGCGGCCGCCTATAAATTCTTAGGTTTAGAGATGATACCATCTG (*Not I*) |
| CDCP1-3’UTR-F1 | CCGCTCGAGCTTGATCCATTCCAGACGCTTTG *(Xho I)* | ATAAGAATGCGGCCGCCTCTGAATCCAGGGCTTGCTG (*Not I*) |
| CDCP1-3’UTR-F2 | CCGCTCGAGCCGCCAACTTCACATTGCTC *(Xho I)* | ATAAGAATGCGGCCGCCGCACAGCCTAAGTTGAGGAG (*Not I*) |
| CRISPR sgRNA |  |  |
| METTL3-sgRNA | CACCGAGACTAGGATGTCGGACACG | AAACCGTGTCCGACATCCTAGTCTC |
| ALKBH5-sgRNA | CACCGGTCCCGGGACAACTATAAGG | AAACCCTTATAGTTGTCCCGGGACC |
| FTO-sgRNA | CACCGGAAGCGCACCCCGACTGCCG | AAACCGGCAGTCGGGGTGCGCTTCC |
| CDCP1-sgRNA | CACCGGTCATGGCCGGCCTGAACTG | AAACCAGTTCAGGCCGGCCATGACC |
| Mutagenesis primers |  |  |
| METTL3-mutant | TTGTGATGGCTGCCCCACCCGCGGATATTCAC | GCGGGTGGGGCAGCCATCACAACTGCAAACTTG |
| CDCP1-3’UTR-mutant1 | AACTTCACATTGCTCAGTGGTCTCATTCTAA | ACCACTGAGCAATGTGAAGTTGGCGG |
| CDCP1-3’UTR-mutant2 | GGTCTCATTCTAAGGGCAAGTCATTGAAAATG | ACTTGCCCTTAGAATGAGACCACTGAGCAATG |
| CDCP1-3’UTR-mutant3 | CCAATCTGGATACAGTCATGTCAGCTCATGT | ACATGACTGTATCCAGATTGGAATTCATCAT |
| Q-PCR Primers |  |  |
| CDCP1-CDS | CTGAACTGCGGGGTCTCTATC | GTCCCCAGCTTTATGAGAACTG |
| CDCP1-3’UTR | CCGCCAACTTCACATTGCTC | CGCACAGCCTAAGTTGAGGA |
| CDCP1-CDS-3’UTR | ACAGCAGGAGGTTTTCCTGG | CAGCCTAAGTTGAGGAGCACA |
| CDCP1-CHECK-3’UTR | CATCAAGAGCTTCGTGGAGC | CGCACAGCCTAAGTTGAGGA |
| SON | CCCTTGTGGTATCATCAGAGACA | CGATGGTACGTCTACAGGCTG |
| HPRT1 | TGACACTGGCAAAACAATGCA | GGTCCTTTTCACCAGCAAGCT |
