# Supplementary material for: Dynamic m6A mRNA methylation reveals the role of METTL3-m6A-CDCP1 signaling axis in chemical carcinogenesis
Source: Oncogene. 2019 Feb 22;38(24):4755–72. doi: 10.1038/s41388-019-0755-0 (PMC6756049; doi:10.1038/s41388-019-0755-0)
Supplement: Supplementary file 15 — Tab.S2 Reagent or Resource [file 41388_2019_755_MOESM15_ESM.docx]

**Table S2. Reagent or Resource**

| REAGENT or RESOURCE | SOURCE | IDENTIFIER |
| --- | --- | --- |
| Antibodies |  |  |
| anti-METTL3(IF) | Abnova | Cat#H00056339-B01P |
| anti-β-actin | Cell Signaling Technology | Cat #4970 |
| anti-GAPDH (14C10) | Cell Signaling Technology | Cat #2118 |
| anti-CDCP1 | Cell Signaling Technology | Cat #4115 |
| anti-rabbit IgG | Cell Signaling Technology | Cat #7074S |
| anti-mouse IgG | Cell Signaling Technology | Cat #7076S |
| anti-ALKBH5 | Sigma-Aldrich | Cat #HPA007196 |
| anti-FLAG | Sigma-Aldrich | Cat #F3165 |
| anti-FTO | PhosphoSolutions | Cat #594-FTO |
| anti-METTL3 | Proteintech | Cat #15073-1-AP |
| anti-YTHDF1 | Proteintech | Cat #17479-1-AP |
| anti-YTHDF2 | Proteintech | Cat #24744-1-AP |
| anti-YTHDF3 | Proteintech | Cat #25537-1-AP |
| anti-m6A | Synaptic Systems | Cat #202 003 |
| Biological Samples |  |  |
| Bladder cancer tissue microarray | Shanghai Outdo Biotech Co., Ltd. | Cat#HBlaU060CS01 |
| Chemicals,Peptides,and Recombinant Proteins | | |
| Actinomycin D | Santa Cruz | Cat #sc-200906 |
| Cadmium chloride | Sigma-Aldrich | Cat #10108-64-2 |
| Fast SYBR Green PCR Master Mix | Thermo Fisher Scientific | Cat #4385612 |
| TransScript All-in-One First-Strand cDNA Synthesis SuperMix for qPCR | Transgen | Cat #AT-341-02 |
| mMESSAGE mMACHINETM T7 | Thermo Fisher Scientific | Cat #AM1344 |
| MEGAscriptTM T7 | Thermo Fisher Scientific | Cat #AM1334 |
| Lipofectamine® 3000 Transfection kit | Thermo Fisher Scientific | Cat #L3000-015 |
| Lipofectamine® RNAiMAX Reagent | Thermo Fisher Scientific | Cat #13778-030 |
| TRIzol Reagent | Thermo Fisher Scientific | Cat #15596018 |
| Critical Commercial Assays | | |
| Flexi® Rabbit Reticulocyte Lysate System | Promega | Cat #L4540 |
| Dual-Luciferase® Reporter Assay System | Promega | Cat #E1960 |
| Magna RIP™RNA-Binding Protein Immunoprecipitation Kit | Millipore | Cat #17-704 |
| Recombinant DNA | | |
| LentiORF pLEX-MCS | Thermo Fisher Scientific | Cat #OHS4735 |
| LentiORF pLEX-MCS-METTL3 | This paper |  |
| LentiORF pLEX-MCS-METTL3-mutant | This paper |  |
| LentiORF pLEX-MCS-ALKBH5 | This paper |  |
| LentiORF pLEX-MCS-FTO | This paper |  |
| LentiORF pLEX-MCS-CDCP1 | This paper |  |
| lentiCRISPR v2 | Addgene | Cat #52961 |
| lentiCRISPR v2-METTL3 | This paper |  |
| lentiCRISPR v2-ALKBH5 | This paper |  |
| lentiCRISPR v2-FTO | This paper |  |
| lentiCRISPR v2-CDCP1 | This paper |  |
| psiCHECK™-2 | Promega | Cat #C8021 |
| psiCHECK™-2-CDCP1-3’UTR-F1 wild type | This paper |  |
| psiCHECK™-2-CDCP1-3’UTR-F1 mutant | This paper |  |
| psiCHECK™-2-CDCP1-3’UTR-F2 wild type | This paper |  |
| psiCHECK™-2-CDCP1-3’UTR-F2 mutant-1 | This paper |  |
| psiCHECK™-2-CDCP1-3’UTR-F2 mutant-2 | This paper |  |
| psiCHECK™-2-CDCP1-3’UTR-F2 mutant-3 | This paper |  |
| pcDNA3-Flag 2AB | gifted from Wang Min |  |
| pcDNA3-Flag 2AB-YTHDF1 | This paper |  |
| pcDNA3-Flag 2AB-YTHDF2 | This paper |  |
| pcDNA3-Flag 2AB-YTHDF3 | This paper |  |
| pcDNA3-Flag 2AB-METTL3 | This paper |  |
| pcDNA3-Flag 2AB-METTL3-mutant | This paper |  |
