# Supplementary material for: Dynamic m6A mRNA methylation reveals the role of METTL3-m6A-CDCP1 signaling axis in chemical carcinogenesis
Source: Oncogene. 2019 Feb 22;38(24):4755–72. doi: 10.1038/s41388-019-0755-0 (PMC6756049; doi:10.1038/s41388-019-0755-0)
Supplement: Supplementary file 16 — Tab. S3 Number of peaks and genes in the control and transformed cells by MeRIP-Seq [file 41388_2019_755_MOESM16_ESM.docx]

**Table S3.** Number of peaks and genes in the control and transformed cells by MeRIP-Seq

| **Cell Lines** | **Number of Peaks** | **Number of Genes** |
| --- | --- | --- |
| SV-HUC-1  Cd-SV-HUC-1  MC-SV-HUC T2  RWPE-1  Cd-RWPE-1  16HBE  NSTC2 | 13623  14364  14470  16284  15237  16239  15898 | 7569  7759  7790  8838  8306  9039  9016 |
