# Supplementary material for: Dynamic m6A mRNA methylation reveals the role of METTL3-m6A-CDCP1 signaling axis in chemical carcinogenesis
Source: Oncogene. 2019 Feb 22;38(24):4755–72. doi: 10.1038/s41388-019-0755-0 (PMC6756049; doi:10.1038/s41388-019-0755-0)
Supplement: Supplementary file 17 — Tab. S4 Number of differential peaks and genes in each set of control to the corresponding transformed cells [file 41388_2019_755_MOESM17_ESM.docx]

**Table S4.** Number of differential peaks and genes in each set of control to the corresponding transformed cells

| **Cell Lines** | **Number of Differential Peaks**  **UP Down** | **Number of Differential Genes**  **UP Down** |
| --- | --- | --- |
| SV-HUC-1 vs. Cd-SV-HUC-1  SV-HUC-1 vs. MC-SV-HUC T2  RWPE-1 vs. Cd-RWPE-1  16HBE vs. NSTC2 | 432 251  730 246  785 478  921 767 | 389 244  659 232  734 444  838 707 |
